# Endemic and threatened birds as surrogates for identifying conservation priority areas and ecological corridors in the America’s most endangered habitat

**DOI:** 10.1038/s41598-024-72948-1

**Published:** 2024-09-20

**Authors:** Thiago da Costa Dias, Luís Fábio Silveira, Mercival Roberto Francisco

**Affiliations:** 1 Programa de Pós-Graduação em Ecologia e Recursos Naturais, Universidade Federal de São Carlos, São Carlos , São Paulo Brazil; 2 Seção de Aves, Museu de Zoologia da Universidade de São Paulo, São Paulo, São Paulo Brazil; 3 Departamento de Ciências Ambientais, Universidade Federal de São Carlos, Campus de Sorocaba, Sorocaba, São Paulo Brazil

**Keywords:** Biodiversity, Conservation biology, Ecological modelling, Macroecology

## Abstract

Investigating multi-taxa macroecological patterns can provide critical insights for spatial conservation planning and landscape management across biodiversity hotspots. The Pernambuco Endemism Center (PEC) is a biogeographic region of the Brazilian Atlantic Forest known to harbor the most threatened habitats in the Americas and a considerable number of recent bird extinctions. Here, we modeled the distribution of 30 threatened forest-dependent birds, 29 of which endemic to the PEC, to reveal key habitats/resources for their survival, identify conservation priority areas, and design ecological corridors. We found variations in the responses of the taxa to landscape characteristics when we addressed these organisms separately and when we grouped them by conservation status. Overall, the environmental variables related to forest quality (e.g., distance to large fragments, distance to the forest edge, percentage of tree cover, percentage of older forests) were important predictors of habitat suitability for the regional threatened avifauna. Additionally, we revealed forest fragments of high ecological importance for the PEC’s threatened birds, and we propose the creation of the *Pernambuco Endemism Center Restoration Arc* (PEC-ARC) that may maximize the investments in conservation and guarantee the connectivity of crucial areas for long-term species survival.

**Supplementary Information:**

The online version contains supplementary material available at 10.1038/s41598-024-72948-1.

## Introduction

Biodiversity has been lost at unprecedented rates because of anthropogenic activities. In the global scenario of intense degradation, tropical forests are of special concern because they keep more than half of the world’s known species, and average deforestation rates of humid primary forests have been approximately 0.3% of the total area per year^1^. In face of the limited monetary resources to invest in conservation, identifying priority areas for conservation has been the most plausible way to preserve as much biodiversity as possible^2,3^. To achieve this purpose, different approaches have been proposed, often based on taxonomic diversity, beta diversity, levels of endemism, and on the presence of rare, threatened, or flag species^3^. Not rarely, however, reserve networks are simply delimited opportunistically in areas not suitable for agriculture, and in these cases, it is often not known whether they really represent the most ecologically relevant areas for the biodiversity of the region^4^.

In megadiverse habitats such as tropical forests, preserving all biodiversity in a target priority area is virtually impossible^5^. Then, a reliable approach is delimiting areas for conservation based on the distribution of threatened taxa^5^. Because individual species can present specific habitat requirements, uncovering areas where the potential distributions of multiple endangered taxa overlap can optimize biodiversity conservation^5^. It means that adequate delimitations of effective conservation areas strongly rely on the knowledge of species distributions, but precise information is scarce for many taxa, especially for those inhabiting megadiverse tropical regions^5,6^. However, in the last decades, species distribution models (SDMs) have provided a tractable way to overcome this limitation^6,7^. These models use mainly climate, topographic, land-use, and vegetation characteristics data from points of confirmed occurrence of the taxa to estimate key habitat requirements, suitable areas, and their potential geographical distribution^8^, being a paramount tool for conservation planning^6^.

When the regions under consideration are drastically disturbed and fragmented, the joint effect of connectivity also must be incorporated in conservation planning, as each independent area may not guarantee the long-term persistence of isolated populations and taxa with larger territorial requirements^9^. Habitat fragmentation imply in habitat reduction for habitat-dependent organisms, decrease movements and gene flow and increase the exposure to the problems of border effect, such as the invasion of alien species and the access by humans^10–15^. Wildlife corridors contribute to organismal dispersal, promoting habitat recolonization and increasing gene flow, therefore reducing the risks of extinction due to demographic and genetic effects^16^. For this reason, when natural corridors are not available, designing protected area networks that facilitate future habitat restoration and reconnections is advised^17^. It is also recommended that corridor implementation must be planed using accurate methods, given the substantial monetary costs for their creation and maintenance^18^.

Many global initiatives to delimit key conservation areas have been proposed based on the occurrence of endemic and/or endangered organisms, such as the hotspots^3^, or the IBAs (Important Bird Areas) for birds^19^. For instance, distribution information of forest-dependent birds was used to develop global maps of priority areas for conservation by attributing impact scores to 5 km cells of forested areas^2^. These scores were determined by the number of species occurring in a target cell (species with limited distribution affecting more) and by the potential impact of the cell loss to the conservation status of the world’s forest-dwelling birds^2^. With this approach, they identified the Atlantic Forest as one of the ecoregions with the highest scores, also highlighting the importance of its northeastern portion (named Pernambuco coastal forests by the authors), ranked among the top 10 regions of special conservation concern for birds on Earth^2^.

Within the Atlantic Forest, the Pernambuco Endemism Center (PEC) is a biogeographic region in northeastern Brazil classified as a hotspot within a hotspot^2,20^. The PEC was home to the modern global bird extinctions in Brazil^21,22^, and undocumented bird extinctions are somehow expected since new species have been recently described and promptly recommended to be listed as threatened (e.g., Alagoas Black-throated Trogon *Trogon muriciensis*^23^). Currently, only about 12% of its original 44,000 km^2^ has remained^24^, and only a small portion is protected^25^. Of the total forest cover, more than 75% is distributed in fragments smaller than 10 km^2^; roughly 90% of the fragments have less than 0.1 km^2^, and about 12% of the remaining forests are higher-quality habitats (i.e., older forest cores in fragments larger than 10 km^2^)^24^.

The dramatic level of fragmentation of the PEC’s forests^24,25^ suggests that the persistence of its forest-dependent avifauna will not rely only on the identification and protection of key conservation areas^22^. Indeed, more energetic conservation actions such as large-scale forest restoration and ecological corridor planning are urgently needed. This region has been considered within the Atlantic Forest hotspot and the Pernambuco coastal forests are placed among the highest-ranked conservation priority areas in the world^2,3^. Additionally, one of the PEC’s conservation unities (Murici Ecological Station) has been recognized as an IBA by Birdlife International due to the presence of critically endangered taxa^26^. However, the identification of key conservation areas and the systematic planning of reserve networks based on accurate methods and criteria were never performed for the PEC.

Here, we used ensemble SDMs for identifying the habitat requirements, distribution, and richness of the endemic and threatened avifauna from the PEC. The products from SDMs were used to calculate the area of suitable landscapes and forests for each bird taxa, as well as the area of suitable habitats located within protected areas. We lastly used spatially-explicit proxies of the probability of occurrence for each taxon from the SDMs together with landscape information to identify conservation priority areas and to plan reserve networks capable of increasing landscape connectivity through forest restoration initiatives. The outcomes of our study may contribute to conservation efforts over the PEC and to guide landscape management for minimizing the wave of bird extinctions that is expected to occur in the PEC.

## Results

**Fig. 1:**
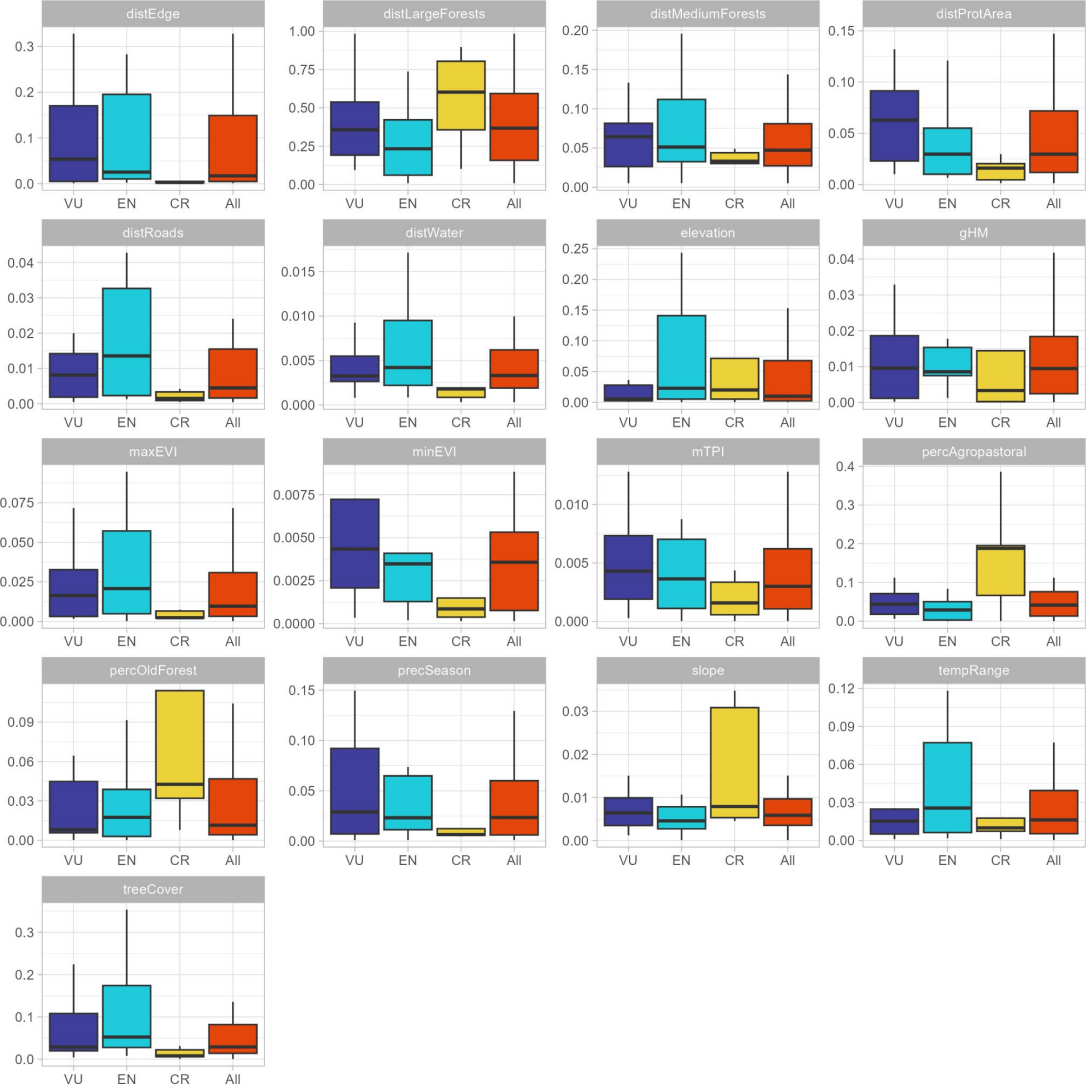
Importance of the different variables used in ensemble models to predict the distribution of threatened forest-dependent avian taxa from the Pernambuco Endemism Center (PEC) in general and to bird assemblages organized by threaten status. Critically Endangered = CR, Endangered = EN, and Vulnerable = VU. Results for Data Deficient (DD) taxa were not shown since this group was represented only by *Hemithraupis flavicollis melanoxantha*.

Ensemble models showed higher accuracy than other single algorithms to predict bird taxa and habitat relationships (Table S5), with TSS accuracies (using testing data) ranging from 0.85 to 1 (Table S5). The variables distance to large fragments, distance to the forest edge, and percentage of tree cover were on average the most important to predict threatened forest-dependent taxa distribution across the PEC (*x* = 0.55, *x* = 0.08, and *x* = 0.08, respectively), but when the taxa were organized by conservation status, the variables distance to large fragments, percentage of older forests, and percentage of agropastoral matrix were more important to predict Critically Endangered taxa when compared to other conservation status’ groups (Fig. 1).

**Fig. 2:**
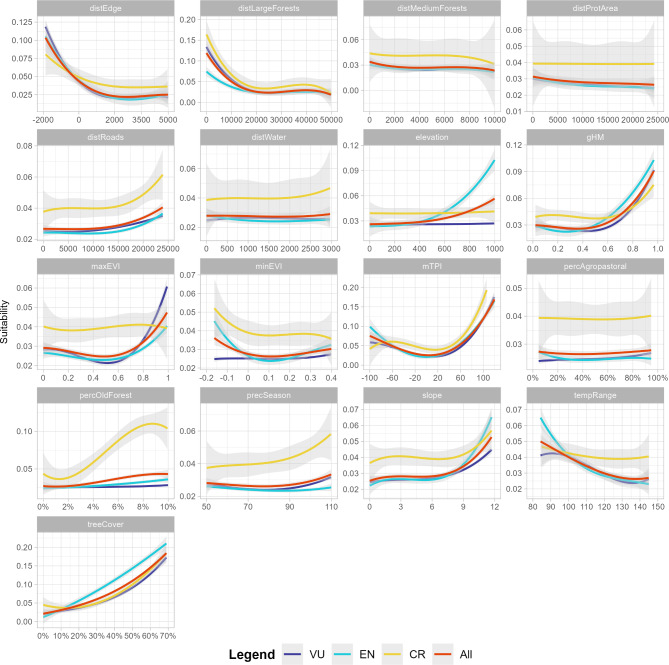
Response curves of threatened forest-dependent taxa from the Pernambuco Endemism Center (PEC) for all birds and assemblages organized by threaten status to 17 environmental variables. Critically Endangered = CR, Endangered = EN, and Vulnerable = VU. Results for Data Deficient (DD) taxa were not shown since this group was only represented by *Hemithraupis flavicollis melanoxantha*.

We found consistent tendencies of habitat selection for the overall bird assemblage and for the birds grouped by level of threat (Fig. 2). Higher suitability was found inside and nearby large fragments, with higher percentages of tree cover, and small variations in temperature across the year, mostly steep and away from roads (Fig. 2). However, Critically Endangered taxa showed preferences for landscapes with higher concentrations of older forests (Fig. 2).

We found that highly threatened taxa were more likely to have reduced landscape and forests available. The threatened forest-dependent avifauna of the PEC had on average 10,556 km^2^ of suitable landscape (protected = 403 km^2^, unprotected = 10,153 km^2^), composed by 2,855 km^2^ of suitable forests (protected = 170 km^2^, unprotected = 2,685 km^2^). The average suitable landscape available for Critically Endangered (CR) taxa was therefore much smaller (remaining = 4,853 km^2^, protected = 186 km^2^, unprotected = 4,667 km^2^), with roughly 1,360 km^2^ of forests (protected = 102 km^2^, unprotected = 1258 km^2^) fitting their habitat requirements. The taxa found to have less available habitat were *Iodopleura pipra leucopygia* (suitable landscape = 42 km^2^, suitable forests = 37 km^2^) and *Megascops alagoensis* (suitable landscape = 76 km^2^, suitable forests = 61 km^2^) (Table 1). Generally, all the taxa had relatively small areas of suitable protected forests (Table 1).

**Table 1:** Remaining habitat (km^2^) for 30 threatened forest-dependent birds of the Pernambuco Endemism Center (PEC), in the northeastern portion of the Brazilian Atlantic Forest.

Taxa	Suit. Land.	Suit. For.	Suit. Land. Prot.	Suit. For. Prot.	Suit. Land. Unprot.	Suit. For. Unprot.	Cons. status
*Automolus lammi*	3,738	1,617	192	126	3,546	1,491	EN
*Caryothraustes brasiliensis* (NE pop.)	2,403	901	75	52	2,328	849	VU
*Cercomacroides laeta sabinoi*	3,855	1,812	199	137	3,656	1,674	VU
*Conopophaga cearae*	4,965	2,046	210	128	4,755	1,918	EN
*Conopophaga melanops nigrifrons*	10,337	3,009	497	199	9,840	2,811	VU
*Dendrocincla taunayi*	494	368	81	69	413	298	EN
*Hemithraupis flavicollis melanoxantha*	1,463	672	116	78	1,347	594	DD
*Hemitriccus griseipectus naumburgae*	8,063	2,807	476	219	7,587	2,588	VU
*Hemitriccus mirandae*	1,794	538	119	66	1,675	472	EN
*Iodopleura pipra leucopygia*	42	37	31	29	11	8.7	EN
*Leptodon forbesi*	10,447	3,208	450	198	9,997	3,010	EN
*Megascops alagoensis*	76	61	17	15	59	46	CR
*Momotus momota marcgravianus*	2,420	1,236	186	118	2,234	1,119	EN
*Myrmoderus ruficauda soror*	3,140	1,286	211	134	2,929	1,152	EN
*Myrmotherula snowi*	744	314	114	84	630	230	CR
*Penelope superciliaris alagoensis*	10,028	3,284	302	152	9,726	3,132	CR
*Phylloscartes ceciliae*	2,742	820	123	93	2,619	727	CR
*Picumnus pernambucensis*	17,501	5,049	791	293	16,710	4,756	VU
*Platyrinchus mystaceus niveigularis*	24,771	5,753	744	286	24,027	5,467	VU
*Pyriglena pernambucensis*	16,906	4,394	482	191	16,424	4,203	VU
*Schiffornis turdina intermedia*	22,944	4,943	769	231	22,175	4,712	VU
*Synallaxis infuscata*	19,604	4,542	462	196	19,142	4,346	EN
*Tangara cyanocephala cearensis*	16,409	4,312	393	192	16,016	4,120	VU
*Tangara fastuosa*	19,582	5,005	786	281	18,796	4,723	VU
*Terenura sicki*	10,675	2,320	375	166	10,300	2,154	CR
*Thalurania watertonii*	21,415	5,251	764	274	20,651	4,977	EN
*Thamnophilus aethiops distans*	14,607	3,961	334	189	14,273	3,772	EN
*Thamnophilus caerulescens pernambucensis*	24,004	5,182	778	264	23,226	4,918	VU
*Xenops minutus alagoanus*	23,207	5,508	1,097	312	22,110	5,197	VU
*Xiphorhynchus atlanticus*	18,307	5,399	923	316	17,384	5,084	VU

Legend: Suit. = Suitable, Land. = Landscape, For. = Forest, Prot. = Protected, Unprot. = Unprotected, Cons. = Conservation.

*Conservation status^21,27^.

**Fig. 3:**
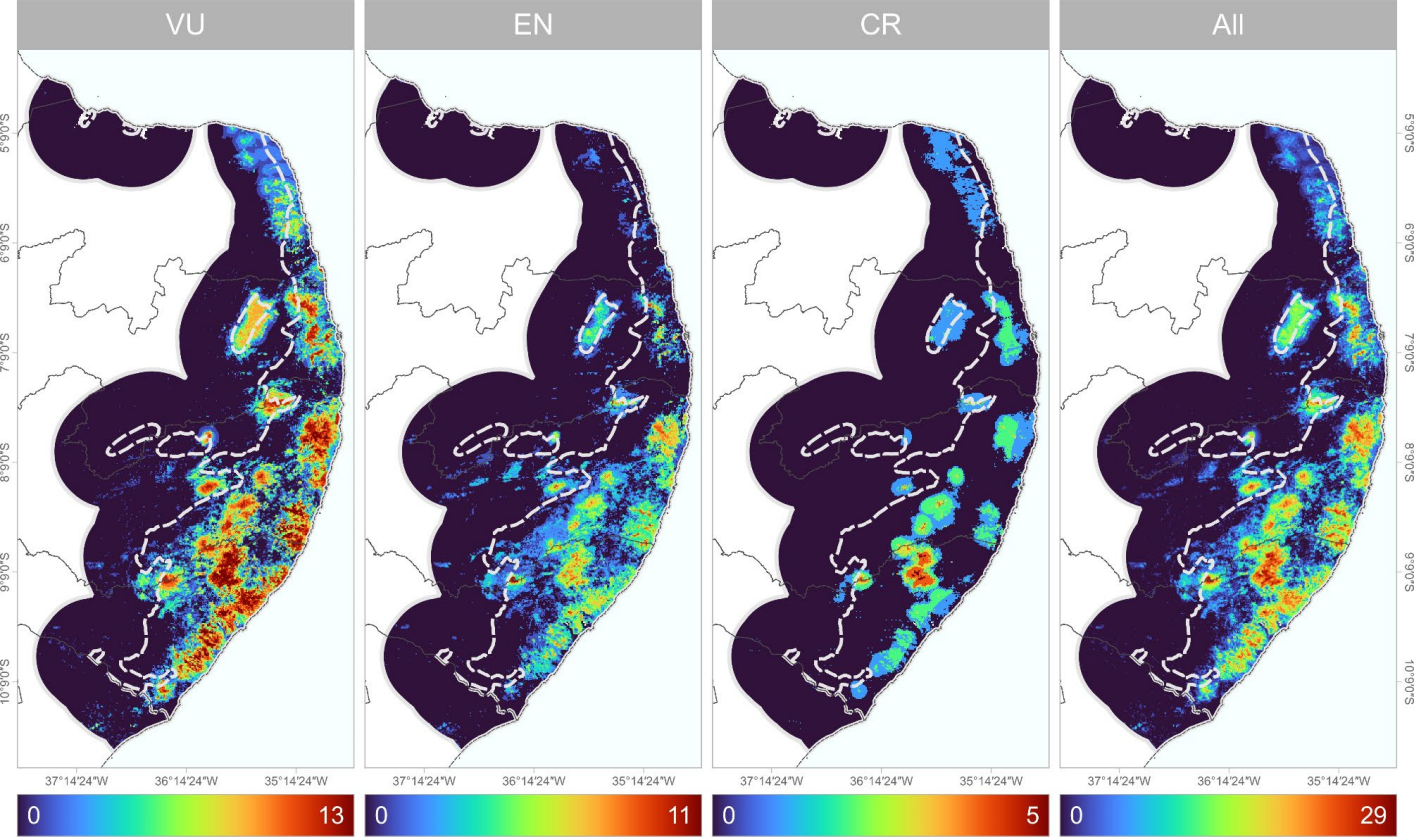
Spatially-explicit patterns of alpha diversity (α-diversity) for threatened forest-dependent bird taxa from the Pernambuco Endemism Center (PEC), in the northeastern portion of the Brazilian Atlantic Forest.

The highest alpha-diversity estimates for threatened forest-dependent birds of the PEC were found in isolated patches, mostly close to the eastern continental border where Atlantic Forest fragments are concentrated (Fig. 3). Areas of higher alpha-diversity of Critically Endangered taxa were found between the states of Alagoas and Pernambuco, specifically close to the protected areas Murici Ecological Station, RPPN Vila d’Água, RPPN Boa Sorte, Pedra Talhada Biological Reserve, RPPN Pedra d’Antas, and RPPN Frei Caneca (Fig. 3).

**Fig. 4:**
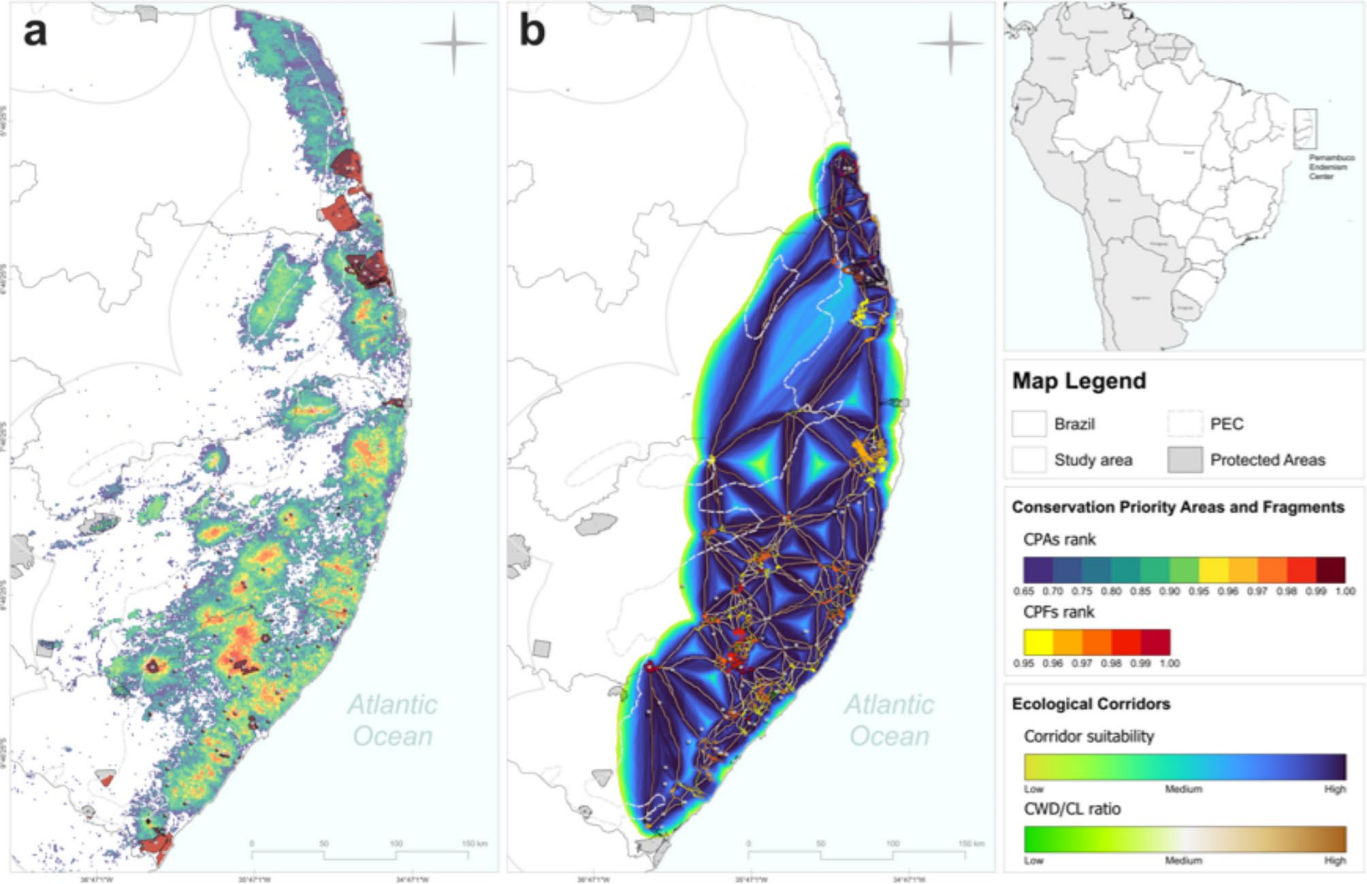
Spatial distribution of (a) Conservation Priority Areas (CPAs), (b) Conservation Priority Fragments (CPFs), and ecological corridors proposed for threatened forest-dependent bird taxa from the Pernambuco Endemism Center (PEC). For the IDs of protected areas, see Table S4.

We identified that the largest CPAs were distributed across Alagoas and Pernambuco states, but some small portions of high conservation value were also found for Paraíba and Rio Grande do Norte (Fig. 4). The portion close to and north of the Murici Ecological Station was the largest conglomerate of high conservation priority areas found for the entire PEC (Fig. 4). Within the CPAs, it was also possible to identify 262 forest patches that were classified as CPFs (> 1 km^2^, conservation priority rank > 0.95) (Fig. 4). The CPFs with the highest rank (> 0.99) were mostly located within protected areas, but several fragments with high conservation values (0.95–0.99) were located outside protected areas (Fig. 4). We propose the creation of 638 ecological corridors between the 262 CPFs, ranging in length from 42 m to about 200 km (*x* = 9.62 km). The CWD/CL ratio ranged from 0.33 to 0.74 (*x* = 0.54). In general, corridors with high CWD/CL ratios were found for the connection between CPFs close to each other, especially for those close to the Murici Ecological Station and its northern portions (Fig. 4).

**Fig. 5:**
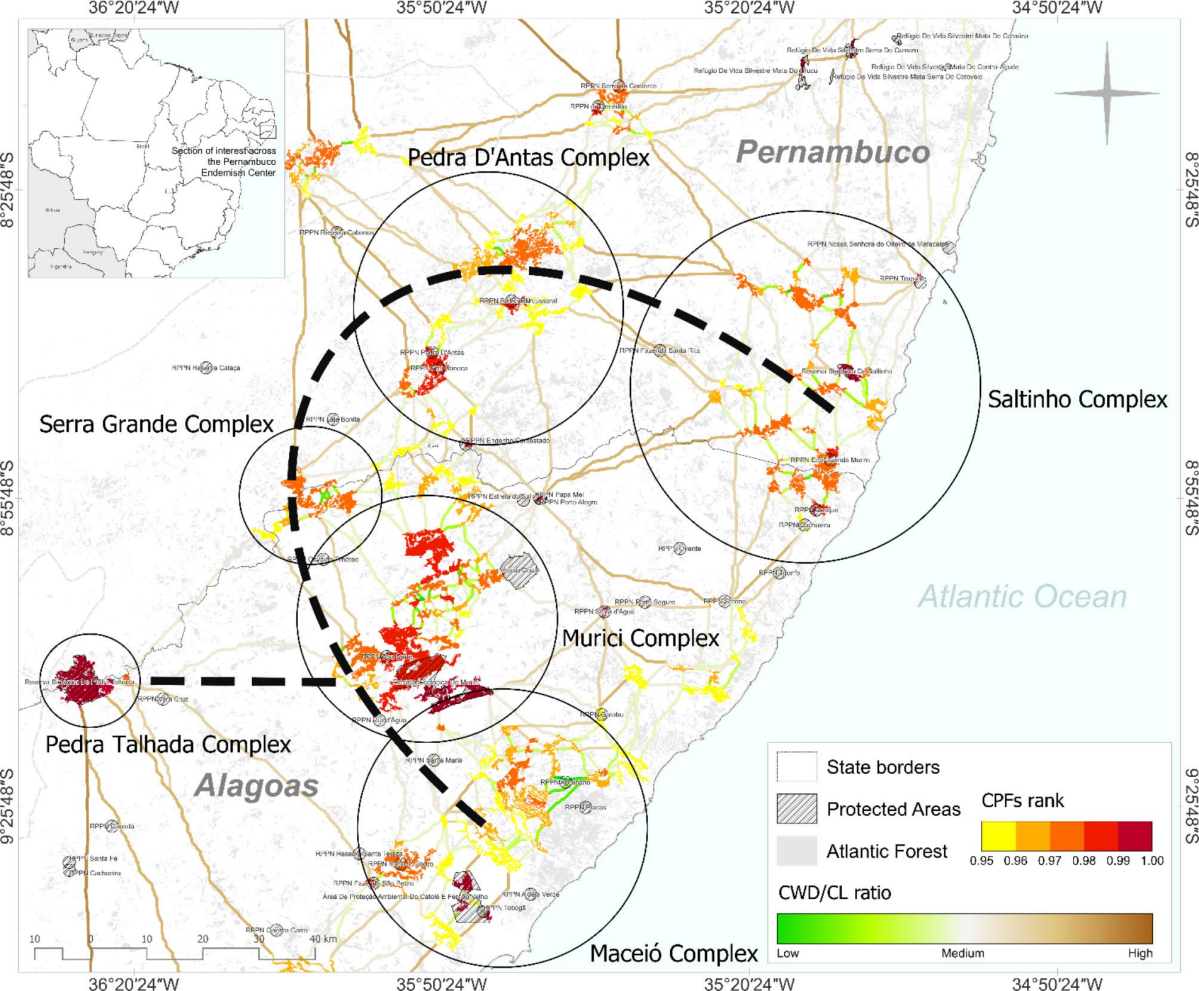
Identification of the *Pernambuco Endemism Center Restoration Arc* (PEC-ARC) with six complexes of Conservation Priority Fragments (CPFs) and corridors with relatively low CWD/CL ratios. Five complexes (Maceió, Murici, Serra Grande, Pedra D’Antas, and Saltinho) show an arc-shaped distribution along northeastern Alagoas and southern Pernambuco states. The connection between Pedra Talhada and Murici complexes is highlighted due to the large forest patches and high conservation importance over these regions. For the IDs of protected areas, see Table S4.

Six complexes with conglomerates of CPFs were identified (Maceió, Murici, Serra Grande, Pedra Talhada, Pedra D’Antas, and Saltinho), within which we detected corridors with low CWD/CL ratios (Fig. 5). On a broader spatial scale, we unrevealed an arc-shaped area suitable for future restoration actions to increase connectivity that involved five out of the six complexes, which we named *Pernambuco Endemism Center Restoration Arc* (PEC-ARC) (Fig. 5).

## Discussion

Ensemble modeling showed high overall accuracy in predicting threatened forest-dependent bird taxa distribution across the PEC, a region where a catastrophic wave of bird extinctions is expected to occur unless urgent conservation actions are developed^28,29^. Landscape features related to forest quality (e.g., distance to edge, percentage of tree cover, and distance to large fragments) were important predictors of habitat suitability for the PEC birds. Higher occupancy probability was generally related to large forest fragments with increased tree cover. Critically Endangered taxa showed relatively higher preferences for areas where older forests were more abundant. It was also possible to identify key areas for bird conservation over the PEC and to propose ecological corridors for reducing the effects of habitat fragmentation, which may therefore increase organismal movement and gene flow. We highlight the importance of the Murici Ecological Station, Pedra Talhada Biological Reserve, and its adjacent areas as bird-diverse regions of top conservation priority, and we highlight the importance of protecting key habitats while increasing connectivity inside the six complexes of CPFs and between them through the implementation of the *Pernambuco Endemism Center Restoration Arc* (PEC-ARC).

The impact of forest characteristics to predict avifauna distribution throughout the PEC was somehow expected since the endemic and threatened bird taxa of the region are mostly forest-dependent^20,22^. Large fragments and older forests also disproportionally affected the presence of Critically Endangered taxa, and their persistence in the future may rely on the protection of large forest remnants mostly composed of older vegetation. This may be challenging since older forest cores in large fragments represent only 12% of the PEC’s remaining forest cover, and they are severely disconected^24^.

Most of the suitable landscapes and forests available for the PEC birds are currently unprotected, and we found that taxa listed under higher threat categories have less suitable areas. The birds with lower availability of suitable landscapes and forests may be prioritized in conservation planning, since they may be more susceptible to extinction due to the restricted distribution of suitable habitats^30,31^. Our findings reinforce the importance of ensuring legal protection to high-quality bird habitats, and because the majority of the PEC’s forests are currently in private lands^32^, encouraging the creation of Private Reserves of Natural Heritage (in Brazil named RPPNs) is a plausible alternative for the region, especially in areas with suitable forests for Critically Endangered taxa and/or high bird diversity.

Although urgently needed, habitat protection alone may be insufficient to prevent future losses of Atlantic Forest birds^33^. For forest-dependent birds, active and passive habitat ecological restoration programs may be a fundamental instrument for increasing landscape connectivity, therefore improving organismal movement, recolonization, and gene flow^16,17^. Based on birds’ responses to landscape features, we identified the *Pernambuco Endemism Center Restoration Arc* (PEC-ARC) where active management aiming at increasing forest connectivity may be especially useful to prevent future global extinctions of the PEC threatened avifauna. The municipalities over this area were among the ones with the highest accumulated deforestation over the last 35 years^24^, which may have contributed with the decline of many the PEC’s bird populations. We also highlight the importance of connecting Murici Ecological Station and Pedra Talhada Biological Reserve, two of the most bird-diverse areas from the region^22^.

Based on our findings, we propose (i) the creation of local restoration programs inside each complex of CPFs (Maceió, Murici, Serra Grande, Pedra D’Antas, Saltinho, and Pedra Talhada), starting with the corridors with low CWD/CL ratio connecting CPFs with higher conservation value, and (ii) the development of reconnection plans over a large spatial scale. One such plan is the Atlantic Forest Restoration Pact (AFRP), an agreement between public and private institutions, governments, companies, the scientific community, and landowners that established the goal of restoring around 10,000 km^2^ of forests in northeastern Atlantic Forest^34^.

We highlight the importance of considering landscape features and the responses of threatened wildlife in conservation planning. Increasing the connectivity of conservation priority fragments through the creation of ecological corridors may be a top-conservation goal to the PEC, which relies on the availability of adequate maps capable of guiding conservation efforts^35^. Increasing surveillance in top-scored habitats (i.e., older forest cores in large fragments with high tree cover) may also increase the protection of threatened bird populations and should be in the scope of conservation managers. Here we identified possible strategies to improve conservation actions for the Pernambuco Endemism Center and its endangered avifauna, which may guide conservation practitioners in their efforts to prevent future extinctions.

## Methods

### Study area

The PEC is a biogeographic region of the Brazilian Atlantic Forest, located north of the São Francisco River in northeastern Brazil. Four major waves of deforestation were responsible for the removal of almost 90% of the PEC’s Atlantic Forest cover during the last centuries^24,32^. The remaining forests are currently threatened by fragmentation (roughly 90% of the fragments are smaller than 0.1 km^2^), edge effects (more than 50% of the forests are within the first 50 m from the border), and rejuvenation (only half of the forests are older than 35 years)^24^. Alarmingly, estimates pointed out that roughly 12% of the remaining forests of the PEC are represented by higher quality habitats (i.e., older forest cores in large fragments)^24^, and that only 8% of the forests show full capacity of carbon storage^36,37^.

We generate a 0.5-degree buffer around the entire Atlantic Forest coverage north from the São Francisco River to define our study area^38^, the extent used to calibrate and evaluate the species distribution models. We choose to include the “Brejos de Altitude” in our region of interest, a set of altitudinal forests, due to the imminent importance of these enclaves to endangered and endemic birds of the PEC, considering potential regions of high ecological importance nearby the PEC borders^39^.

### Occurrence dataset

We downloaded occurrence data for 34 threatened forest-dependent bird taxa occurring in the PEC (Table S1), and except for *Conopophaga cearae*, all of them are endemic to this endemism area. Taxa occurrence data were downloaded from the list of specimens housed at Museu de Zoologia da Universidade de São Paulo (MZUSP); the Global Biodiversity Information Facility – GBIF (https://www.gbif.org); the ATLANTIC BIRDS dataset^40^; and from the Brazilian Biodiversity Extinction Risk Assessment System – SALVE^41^. GBIF represents the largest and most widely used biodiversity dataset of species occurrence in the world^42^. ATLANTIC BIRDS is the most complete dataset on Brazilian Atlantic Forest birds’ occurrences, which compiled unpublished reports and published data from museum collections, literature, and other online data sources^40^. The SALVE system is a consolidated database developed by the Chico Mendes Institute for Biodiversity Conservation – ICMBio to facilitate the process of extinction risk assessment of Brazilian species^41^. Taxa were searched by their scientific names and protonyms, and in the case of subspecies, we also searched for the full-species name. We assessed and downloaded GBIF data using the ‘rgbif’ package^43^ in R environment^44^, ATLANTIC BIRDS dataset through the paper Supplementary Material^40^, and SALVE data from https://sicae.sisicmbio.icmbio.gov.br.

Using the ‘CoordinateCleaner’ package^45^ in R, we applied the automated cleaning framework to filter our mixed dataset^45^. We first removed occurrences with no coordinates, within marine areas, coordinate-country mismatches, occurrences assigned to political units’ centroids (country, state, and municipality), outlier coordinates, and coordinates assigned to research institutions. We also removed data with low coordinate precision (larger than 1 km due to the spatial resolution of the environmental variables used for SDMs), individual counts smaller than one and larger than 99, and data collected before 1945 (we only kept data after the end of the Second World War due to the common imprecision of old records). Occurrences outside our study area and in non-vegetated areas (according to the 2020 land use and land cover layer from MapBiomas^46^) were also removed. Lastly, for each taxon, spatial duplicates were removed, and coordinates within 1 km from each other were deleted prioritizing the most recent occurrence in a 1 km buffer to minimize spatial autocorrelation. After this process, organisms with less than five occurrences were removed from further analysis, which resulted in a list of 30 from the 34 original taxa.

### Environmental variables

We used Google Earth Engine^47^ to assess and generate 31 environmental variables representative of the PEC’s terrain, climate, forest cover, biomass, human impacts, and water bodies (Table S2). For variables related to the PEC forest cover, we first assessed and downloaded the 2020 MapBiomas land use and land cover layer using Google Earth Engine and used the ‘bfastspatial’ package^48^ in R to calculate fragment size and to assign them individual identification numbers. All layers were rescaled to 1 km spatial resolution, and we assessed correlation using Pearson’s correlation test and removed variables with *r* > 0.7^49^ (Figure S1). Multicollinearity was then assessed using the Variation Inflation Factor (VIF), and we continuously removed variables with higher VIF values until all variables showed VIF < 5^50^ (Table S3). After this procedure, 17 variables were kept for modeling species distribution: distEdge, distLargeForests, distMediumForests, distProtArea, distRoads, distWater, elevation, gHM, maxEVI, minEVI, mTPI, percAgropastoral, percOldForest, precSeason, slope, tempRange, and treeCover (see Appendix 2 for the full description of the variables). We used the same set of environmental variables for all species since correlation and multicollinearity were assessed using the whole landscape, instead of individual species, which also allowed grouping the species’ responses to variables according to their conservation status^51–53^.

### Species distribution modeling

Due to the great availability of algorithms used for modeling species distribution and the variation in their predictive performance across species, regions, and applications, authors have suggested that combining predictions from different models (ensemble modeling) may be useful and produce more reliable results^54^. We modeled endemic and endangered bird taxa distribution using the ‘biomod2’ package^55^ in R to build ensembles of SDMs. We built models using five widely-used algorithms (generalized linear models – GLM, generalized boosted models – GBM, classification tree analysis – CTA, artificial neural networks – ANN, and maximum entropy – MAXENT), chosen due to its advantages in terms of optimizing models with high predictive performance while reducing computation time^56^. For each taxon, we created three random sets of pseudo-absences with 10,000 background points, generated with a minimum point-to-point distance of 1 km^53^. Occurrences and background points were equally down weighted by setting the prevalence parameter to 0.546. We ran 100 replications using 80% of data for calibration and 20% for evaluation, totalizing 1,500 models for each taxon (3 sets of pseudo-absences x 5 algorithms x 100 replications). These models were individually evaluated based on the True Statistic Skill (TSS) and the Receiver Operating-Characteristic (ROC)^57,58^.

The final ensemble models were built by calculating the proportionally weighted sum of probabilities (weights were attributed proportionally to the value of the evaluation score TSS) across predictions of models with TSS > 0.8^60^. From the ensemble models, we extracted the (i) variables’ importance, (ii) response curves for the environmental variables, (iii) habitat suitability layers, (iv) binary layers representatives of the taxa distribution, (v) area of the suitable landscape, suitable forests using the MapBiomas land use and land cover layer, and distribution within protected areas using the World Database on Protected Areas – WDPA^60^, and (vi) layers on the endemic and/or endangered birds’ alpha diversity (*α*-diversity), generated by summing all binary occurrence layers. When convenient, results were aggregated by taxa conservation status into Data Deficient (DD), Vulnerable (VU), Endangered (EN), and Critically Endangered (CR)^21,27^.

### Conservation priority areas

We used Zonation 5 v1.0 to identify Conservation Priority Areas (CPAs) through a hierarchical-driven approach^61^. This software implements an analysis that first assumes that all cells over the landscape must be prioritized for conservation and then it removes pixels gradually based on the least overall loss for biodiversity subject to what remains in the environment^62^. We performed the identification of CPAs based on the Core Area Zonation rule for pixel removal (CAZ1), which assigns higher values to areas where highly-weighted taxa occur^61^. The CAZ1 rule was used to assign higher values for taxa facing more threats (i.e., critically endangered).

We included the habitat suitability layers in Zonation by first assigning zero to all non-occurrence pixels (below the individual TSS threshold) based on the binary occurrence layers from the ensemble modeling. Those layers were then weighted according to the taxon conservation status (Data deficient [DD] = 1, Vulnerable [VU] = 2, Endangered [EN] = 3, and Critically Endangered [CR] = 4)^49,64^. We also included the Global Human Modification (gHM) layer as a proxy of the landscape ecological condition^66^, penalizing pixels in regions of higher human influence^62,63^ since they may represent areas of low conservation concern for the PEC’s avifauna, as well as built-up areas.

Finally, we merged the World Database on Protected Areas – WDPA^60^ to a buffer of 1 km around the coordinates of each federal and state private reserve of natural heritage (RPPNs) in the PEC (for IDs of protected areas, see Table S4). Protected areas were then included as a hierarchical mask in our analysis, forcing Zonation to consider the pre-existence of protected areas and then include non-protected cells of ecological importance to generate the CPAs^49^.

### Corridor planning

We used the Linkage Pathways Tool from the Linkage Mapper Toolbox in ArcGIS^65^ for proposing ecological corridors to connect forest fragments of high conservation value for the PEC’s endemic and endangered avifauna. This tool generates the least-cost pathways between pre-determined areas by considering the surface resistance to animal movement/dispersal^67^. For the definition of the areas to be connected, we first used the 2020 land use and land cover layer from MapBiomas to select Atlantic Forest fragments larger than 1111 pixels (approximately 1 km^2^), since the larger fragments may present higher species richness^67^. For each of these fragments, we extracted the mean value derived from the Zonation rank map and removed all fragments with values lower than 0.95. The final Conservation Priority Fragments (CPFs) layer was composed of fragments larger than 1 km^2^ with an average conservation priority value > 95. After the identification of CPFs, we generated a resistance surface layer for corridor modeling by considering landscape features and the responses of bird taxa to the PEC environment. We used five layers representative of landscape features, which were rescaled to range from 0 (non-resistance) to 1 (maximum resistance). The first layer represents the Atlantic Forest fragments from the 2020 MapBiomas land use and land cover (zero resistance = forest, one resistance = non-forest). The second was the rescaled inverse of the tree canopy coverage^68^ (higher resistances for lower tree cover). We also calculated the Euclidean distance to Atlantic Forest fragments and assigned higher resistances to areas further from forest patches. To prioritize corridors that would ensure the restoration of the existing legal debt of the PEC’s Atlantic Forest, we generated a layer with resistance values set to zero in riparian areas of permanent preservation (APPs) without forests, and to one the areas outside or within APPs but already filled with forests^69^. We lastly added a proxy of barriers (water bodies from the National Water and Sanitation Agency – ANA, and roads from Brazil’s National Transport Infrastructure Department - DNIT) to taxa movement across the PEC (resistance in barriers set to one, and in all other features of the landscape set to zero). The final landscape resistance layer was obtained by calculating the mean of the five abovementioned layers.

Posterior to calculating landscape resistance, we generated resistance layers according to taxa responses to the environment by applying the following equation to habitat suitability layers (hs) derived from the ensemble SDMs^70^:

1 $$\left( {\left( {hs - \hbox{max} \left( {hs} \right)} \right)* - 1} \right)+\hbox{min} \left( {hs} \right)$$

All bird taxa resistance layers were averaged. Lastly, landscape resistance and taxa resistance layers were summed and divided by two to obtain the final resistance layer, which was included in the Linkage Pathways Tool for corridor planning. We lastly calculated the cost-weighted distance/corridor length (CWD/CL) ratio as a proxy of the adversity for bird taxa to disperse and landscape resistance to forest restoration through the proposed corridors^71^.

## Electronic supplementary material

Below is the link to the electronic supplementary material.

Supplementary Material 1

## Data Availability

All data generated or analyzed during this study are included in this published article (and its Supplementary Information files) and are fully available in Figshare data repository through the link.
