# Supplementary material for: Endemic and threatened birds as surrogates for identifying conservation priority areas and ecological corridors in the America’s most endangered habitat
Source: Sci Rep. 2024 Sep 20;14:21923. doi: 10.1038/s41598-024-72948-1 (PMC11412977; doi:10.1038/s41598-024-72948-1)
Supplement: Supplementary file 1 — Supplementary Material 1 [file 41598_2024_72948_MOESM1_ESM.pdf]

## APPENDIX 1. ENDANGERED AND ENDEMIC BIRD TAXA FROM THE PERNAMBUCO ENDEMISM CENTER

Table S1. Bird taxa from the Pernambuco Endemism Center (PEC) assessed during our study. The taxa in bold represents species removed from the analysis due to the low number of occurrence points.

| ID | Scientific name | Distribution | Cons. status | | Occ. points |
|---|---|---|---|---|---|
| | | | IUCN | ICMBio, MMA | |
| 1 | *Automolus lammi* | PEC | EN | EN | 39 |
| 2 | *Caryothraustes brasiliensis* (NE pop.) | PEC | - | VU | 14 |
| 3 | *Cercomacroides laeta sabinoi* | PEC | - | VU | 30 |
| 4 | *Conopophaga cearae* | AF | NT | EN | 26 |
| 5 | *Conopophaga melanops nigrifrons* | PEC | - | VU | 26 |
| 6 | *Dendrocincla taunayi* | PEC | - | EN | 9 |
| 7 | *Hemithraupis flavicollis melanoxantha* | PEC | - | DD | 16 |
| 8 | *Hemitriccus griseipectus naumburgae* | PEC | - | VU | 58 |
| 9 | *Hemitriccus mirandae* | PEC | VU | EN | 13 |
| 10 | *Iodopleura pipra leucopygia* | PEC | - | EN | 6 |
| 11 | *Leptodon forbesi* | PEC | EN | EN | 57 |
| 12 | *Megascops alagoensis* | PEC | - | CR** | 7 |
| 13 | *Momotus momota marcgravianus* | PEC | - | EN | 12 |
| 14 | *Myrmoderus ruficauda soror* | PEC | - | EN* | 41 |
| 15 | *Myrmotherula snowi* | PEC | CR | CR | 16 |
| **16** | ***Odontophorus capueira plumbeicollis*** | **PEC** | **-** | **CR** | **3** |
| 17 | *Penelope superciliaris alagoensis* | PEC | - | CR | 16 |
| **18** | ***Phaethornis margarettae camargoi*** | **PEC** | **-** | **EN** | **3** |
| 19 | *Phylloscartes ceciliae* | PEC | CR | CR | 42 |
| 20 | *Picumnus pernambucensis* | PEC | - | VU | 38 |
| 21 | *Platyrinchus mystaceus niveigularis* | PEC | LC | VU | 27 |
| 22 | *Pyriglena pernambucensis* | PEC | - | VU | 22 |
| 23 | *Schiffornis turdina intermedia* | PEC | - | VU | 18 |
| **24** | ***Sclerurus caudacutus caligineus*** | **PEC** | **-** | **CR** | **4** |
| 25 | *Synallaxis infuscata* | PEC | EN | EN | 89 |
| 26 | *Tangara cyanocephala cearensis* | PEC | - | VU | 16 |
| 27 | *Tangara fastuosa* | PEC | VU | VU | 63 |

| | | | | | |
|---|---|---|---|---|---|
| 28 | *Terenura sicki* | PEC | CR | CR | 41 |
| 29 | *Thalurania watertonii* | PEC | EN | EN | 40 |
| 30 | *Thamnophilus aethiops distans* | PEC | - | EN | 20 |
| 31 | *Thamnophilus caerulescens pernambucensis* | PEC | - | VU | 24 |
| **32** | ***Trogon muriciensis*** | **PEC** | **-** | **CR*** ** | **1** |
| 33 | *Xenops minutus alagoanus* | PEC | LC | VU | 51 |
| 34 | *Xiphorhynchus atlanticus* | PEC | - | VU | 45 |

Distribution from specimens housed at MZUSP, Roda *et al*. (2011), and Dantas *et al*. (2021). Distribution: PEC = PEC-endemic, AF = Atlantic Forest-endemic

*Risk assessed at higher taxonomic levels

**Conservation status suggested by Dantas *et al*. (2021)

***Conservation status suggested by Dickens *et al.* (2021)

## References


Automolus lammi J.T.Zimmer, 1947 in GBIF Secretariat (2023). GBIF Backbone Taxonomy. Checklist dataset https://doi.org/10.15468/39omei accessed via GBIF.org on 2024-08-06.

Caryothraustes canadensis subsp. brasiliensis Cabanis, 1851 in GBIF Secretariat (2023). GBIF Backbone Taxonomy. Checklist dataset https://doi.org/10.15468/39omei accessed via GBIF.org on 2024-08-06.

Cercomacroides laeta subsp. sabinoi (Pinto, 1939) in GBIF Secretariat (2023). GBIF Backbone Taxonomy. Checklist dataset https://doi.org/10.15468/39omei accessed via GBIF.org on 2024-08-06.

Conopophaga cearae Cory, 1916 in GBIF Secretariat (2023). GBIF Backbone Taxonomy. Checklist dataset https://doi.org/10.15468/39omei accessed via GBIF.org on 2024-08-06.

Conopophaga melanops subsp. nigrifrons Pinto, 1954 in GBIF Secretariat (2023). GBIF Backbone Taxonomy. Checklist dataset https://doi.org/10.15468/39omei accessed via GBIF.org on 2024-08-06.

Dantas, S. M., Weckstein, J. D., Bates, J., Oliveira, J. N., Catanach, T. A., & Aleixo, A. (2021). Multi-character taxonomic review, systematics, and biogeography of the Black-capped/Tawny-bellied Screech Owl (*Megascops atricapilla*-M. watsonii) complex (Aves: Strigidae). Zootaxa, 4949(3), 401-444.

Dendrocincla G.R.Gray, 1840 in GBIF Secretariat (2023). GBIF Backbone Taxonomy. Checklist dataset https://doi.org/10.15468/39omei accessed via GBIF.org on 2024-08-06.

Dickens, J. K., Bitton, P.-P., Bravo, G. A., & Silveira, L. F. (2021). Species limits, patterns of secondary contact and a new species in the Trogon rufus complex (Aves: Trogonidae). Zoological Journal of the Linnean Society, 193(2), 499–540.

Hemithraupis flavicollis subsp. melanoxantha (M.H.K.Lichtenstein, 1823) in GBIF Secretariat (2023). GBIF Backbone Taxonomy. Checklist dataset https://doi.org/10.15468/39omei accessed via GBIF.org on 2024-08-06.

Hemitriccus griseipectus subsp. naumburgae (Zimmer, 1945) in GBIF Secretariat (2023). GBIF Backbone Taxonomy. Checklist dataset https://doi.org/10.15468/39omei accessed via GBIF.org on 2024-08-06.

Hemitriccus mirandae (E.Snethlage, 1925) in GBIF Secretariat (2023). GBIF Backbone Taxonomy. Checklist dataset https://doi.org/10.15468/39omei accessed via GBIF.org on 2024-08-06.

ICMBio. (2018). Livro Vermelho da Fauna Brasileira Ameaçada De Extinção: volume III – Aves.

Iodopleura pipra subsp. leucopygia Salvin, 1885 in GBIF Secretariat (2023). GBIF Backbone Taxonomy. Checklist dataset https://doi.org/10.15468/39omei accessed via GBIF.org on 2024-08-06.

IUCN. (2022). The IUCN Red List of Threatened Species. Version 2022-2.

Lepage, D., & Warnier, J. (2014). The Peters' check-list of the birds of the world (1931–1987) database. Accessed On, 11(09), 2018.

Leptodon forbesi (Swann, 1922) in GBIF Secretariat (2023). GBIF Backbone Taxonomy. Checklist dataset https://doi.org/10.15468/39omei accessed via GBIF.org on 2024-08-06.

Lima, R. D., Silveira, L. F., Lemos, R. C. de A., Lobo-Araújo, L. W., Andrade, A. B. de, Francisco, M. R., & Efe, M. A. (2022). An annotated avian inventory of the Brazilian state of Alagoas, one of the world's most threatened avifauna. Papéis Avulsos de Zoologia, 62.

Megascops alagoensis Dantas, Weckstein, Bates, Oliveira, Catanach & Aleixo, 2021 in GBIF Secretariat (2023). GBIF Backbone Taxonomy. Checklist dataset https://doi.org/10.15468/39omei accessed via GBIF.org on 2024-08-06.

MMA. (2022). Portaria MMA no 148, de 7 de junho de 2022. Altera os Anexos da Portaria no 443, de 17 de dezembro de 2014, da Portaria no 444, de 17 de dezembro de 2014, e da Portaria no 445, de 17 de dezembro de 2014, referentes à atualização da Lista Nacional de Espécies Ameaçadas de Extinção (pp. 1–116). Ministério do Meio Ambiente. https://www.icmbio.gov.br/cepsul/images/stories/legislacao/Portaria/2020/P_mma_148_2022_altera_anexos_P_mma_443_444_445_2014_atualiza_especies_ameacadas_extincao.pdf

Momotus momota subsp. marcgravianus Pinto & Camargo, 1961 in GBIF Secretariat (2023). GBIF Backbone Taxonomy. Checklist dataset https://doi.org/10.15468/39omei accessed via GBIF.org on 2024-08-06.

Myrmoderus ruficauda subsp. soror (Pinto, 1940) in GBIF Secretariat (2023). GBIF Backbone Taxonomy. Checklist dataset https://doi.org/10.15468/39omei accessed via GBIF.org on 2024-08-06.

Myrmotherula snowi Teixeira & Gonzaga, 1985 in GBIF Secretariat (2023). GBIF Backbone Taxonomy. Checklist dataset https://doi.org/10.15468/39omei accessed via GBIF.org on 2024-08-06.

Odontophorus capueira subsp. plumbeicollis Cory, 1915 in

Pacheco, J. F., Silveira, L. F., Aleixo, A., Agne, C. E., Bencke, G. A., Bravo, G. A., ... & de Q. Piacentini, V. (2021). Annotated checklist of the birds of Brazil by the Brazilian Ornithological Records Committee—second edition. Ornithology Research, 29(2), 94-105.

Penelope superciliaris subsp. alagoensis Nardelli, 1993 in GBIF Secretariat (2023). GBIF Backbone Taxonomy. Checklist dataset https://doi.org/10.15468/39omei accessed via GBIF.org on 2024-08-06.

Phaethornis Swainson, 1827 in GBIF Secretariat (2023). GBIF Backbone Taxonomy. Checklist dataset https://doi.org/10.15468/39omei accessed via GBIF.org on 2024-08-06.

Phylloscartes ceciliae Teixeira, 1987 in GBIF Secretariat (2023). GBIF Backbone Taxonomy. Checklist dataset https://doi.org/10.15468/39omei accessed via GBIF.org on 2024-08-06.

Picumnus Temminck, 1825 in GBIF Secretariat (2023). GBIF Backbone Taxonomy. Checklist dataset https://doi.org/10.15468/39omei accessed via GBIF.org on 2024-08-06.

Platyrinchus mystaceus subsp. niveigularis Pinto, 1954 in GBIF Secretariat (2023). GBIF Backbone Taxonomy. Checklist dataset https://doi.org/10.15468/39omei accessed via GBIF.org on 2024-08-06.

Pyriglena Cabanis, 1847 in GBIF Secretariat (2023). GBIF Backbone Taxonomy. Checklist dataset https://doi.org/10.15468/39omei accessed via GBIF.org on 2024-08-06.

Roda, S. A., Pereira, G. A., & Albano, C. (2011). Conservação de Aves endêmicas e ameaçadas do Centro de Endemismo Pernambuco. Editora Universitária UFPE, Recife, 79pp.

Schiffornis turdina subsp. intermedia Pinto, 1954 in GBIF Secretariat (2023). GBIF Backbone Taxonomy. Checklist dataset https://doi.org/10.15468/39omei accessed via GBIF.org on 2024-08-06.

Sclerurus caudacutus subsp. caligineus Pinto, 1954 in GBIF Secretariat (2023). GBIF Backbone Taxonomy. Checklist dataset https://doi.org/10.15468/39omei accessed via GBIF.org on 2024-08-06.

Synallaxis infuscata Pinto, 1950 in GBIF Secretariat (2023). GBIF Backbone Taxonomy. Checklist dataset https://doi.org/10.15468/39omei accessed via GBIF.org on 2024-08-06.

Tangara cyanocephala subsp. cearensis Cory, 1916 in GBIF Secretariat (2023). GBIF Backbone Taxonomy. Checklist dataset https://doi.org/10.15468/39omei accessed via GBIF.org on 2024-08-06.

Tangara fastuosa (R.Lesson, 1831) in GBIF Secretariat (2023). GBIF Backbone Taxonomy. Checklist dataset https://doi.org/10.15468/39omei accessed via GBIF.org on 2024-08-06.

Terenura sicki Teixeira & Gonzaga, 1983 in GBIF Secretariat (2023). GBIF Backbone Taxonomy. Checklist dataset https://doi.org/10.15468/39omei accessed via GBIF.org on 2024-08-06.

Thalurania watertonii (Bourcier, 1847) in GBIF Secretariat (2023). GBIF Backbone Taxonomy. Checklist dataset https://doi.org/10.15468/39omei accessed via GBIF.org on 2024-08-06.

Thamnophilus aethiops subsp. distans Pinto, 1954 in GBIF Secretariat (2023). GBIF Backbone Taxonomy. Checklist dataset https://doi.org/10.15468/39omei accessed via GBIF.org on 2024-08-06.

Thamnophilus caerulescens subsp. pernambucensis Naumburg, 1937 in GBIF Secretariat (2023). GBIF Backbone Taxonomy. Checklist dataset https://doi.org/10.15468/39omei accessed via GBIF.org on 2024-08-06.

Trogon muriciensis Dickens, Bitton, Bravo & Silveira in GBIF Secretariat (2023). GBIF Backbone Taxonomy. Checklist dataset https://doi.org/10.15468/39omei accessed via GBIF.org on 2024-08-06.

Xenops minutus subsp. alagoanus in GBIF Secretariat (2023). GBIF Backbone Taxonomy. Checklist dataset https://doi.org/10.15468/39omei accessed via GBIF.org on 2024-08-06.

Xiphorhynchus atlanticus (Cory, 1916) in GBIF Secretariat (2023). GBIF Backbone Taxonomy. Checklist dataset https://doi.org/10.15468/39omei accessed via GBIF.org on 2024-08-06.

## APPENDIX 2. ENVIRONMENTAL VARIABLES REPRESENTING THE PERNAMBUCO ENDEMISM CENTER HABITATS AND COMPARISON OF GLOBAL HUMAN MODIFICATION BETWEEN PRESENCE AND BACKGROUND POINTS

Table S2. Environmental variables used for species distribution modeling.

| Group | Included | Variable | Dataset Provider | Description |
|---|---|---|---|---|
| Terrain | Yes | elevation | NASA / USGS / JPL-Caltech | Digital elevation data. |
| | Yes | slope | NASA / USGS / JPL-Caltech | Represents the steepness of the ground surface. |
| | Yes | mTPI | Conservation Science Partners | Multi-Scale Topographic Position Index, calculated using elevation subtracted by the mean elevation within a neighborhood. |
| | No | topoDiversity | Conservation Science Partners | Topographic diversity represents the variety of temperature and moisture conditions. |
| Climate | No | tempMean | University of California, Berkeley | WorldClim V1 Bioclim, annual mean temperature. |
| | No | tempSeason | University of California, Berkeley | WorldClim V1 Bioclim, annual temperature seasonality. |
| | No | tempMax | University of California, Berkeley | WorldClim V1 Bioclim, maximum temperature in the hottest month. |
| | No | tempMin | University of California, Berkeley | WorldClim V1 Bioclim, minimum temperature in the coldest month. |
| | Yes | tempRange | University of California, Berkeley | WorldClim V1 Bioclim, annual temperature range. |
| | No | precAnnual | University of California, Berkeley | WorldClim V1 Bioclim, accumulated annual precipitation. |
| | No | precWet | University of California, Berkeley | WorldClim V1 Bioclim, precipitation in the wettest month. |
| | No | precDry | University of California, Berkeley | WorldClim V1 Bioclim, precipitation in the driest month. |
| | Yes | precSeason | University of California, Berkeley | WorldClim V1 Bioclim, annual precipitation seasonality. |
| Human | Yes | gHM | Conservation Science Partners | Global Human Modification dataset, cumulative measure of human modification. |
| | Yes | distProtArea | UN Environment World Conservation Monitoring Centre (UNEP-WCMC) / Protected Planet | Euclidean distance to protected areas generated using the World Database on Protected Areas. |
| | Yes | distRoads | Brazil's national transport infrastructure department (DNIT) | Euclidean distance to roads generated using federal and state road data from DNIT. |
| | Yes | percAgropastoral | MapBiomas Collection 6.0 | Percentage of the agropastoral matrix in a 1-km radius buffer. |
| Forest | No | percOtherForests | MapBiomas Collection 6.0 | Percentage of non-Atlantic Forest in a 1-km radius buffer. |
| | Yes | distLargeForests | MapBiomas Collection 6.0 | Euclidean distance to > 10 km² Atlantic Forest fragments. |
| | Yes | distMediumForests | MapBiomas Collection 6.0 | Euclidean distance to 1 – 10 km² Atlantic Forest fragments. |
| | No | distSmallForests | MapBiomas Collection 6.0 | Euclidean distance to < 1 km² Atlantic Forest fragments. |

| | | | | |
|---|---|---|---|---|
| | No | nFrag | MapBiomas Collection 6.0 | Number of Atlantic Forest fragments in a 1-km radius buffer. |
| | Yes | distEdge | MapBiomas Collection 6.0 | Euclidean distance to Atlantic Forest borders. Negative values represent distances from the border to the forest core. Positive values represent distances away from the forest borders into the matrix. |
| | Yes | treeCover | NASA LP DAAC at the USGS EROS Center | The percentage of a pixel covered by trees. |
| | Yes | percOldForest | MapBiomas Collection 6.0 | Percentage of older (> 35 years) Atlantic Forest in a 1-km radius buffer. |
| | No | percYoungForest | MapBiomas Collection 6.0 | Percentage of younger (< 35 years) Atlantic Forest in a 1-km radius buffer. |
| Biomass | No | meanEVI | NASA LP DAAC at the USGS EROS Center | 1-km mean EVI (2018-01-01 - 2022-12-31). |
| | Yes | minEVI | NASA LP DAAC at the USGS EROS Center | 1-km minimum EVI (2018-01-01 - 2022-12-31). |
| | Yes | maxEVI | NASA LP DAAC at the USGS EROS Center | 1-km maximum EVI (2018-01-01 - 2022-12-31). |
| | No | diffEVI | NASA LP DAAC at the USGS EROS Center | Difference between maximum EVI and minimum EVI. |
| Other | Yes | distWater | Brazil's National Water and Sanitation Agency | Euclidean distance to watercourses and water bodies. |

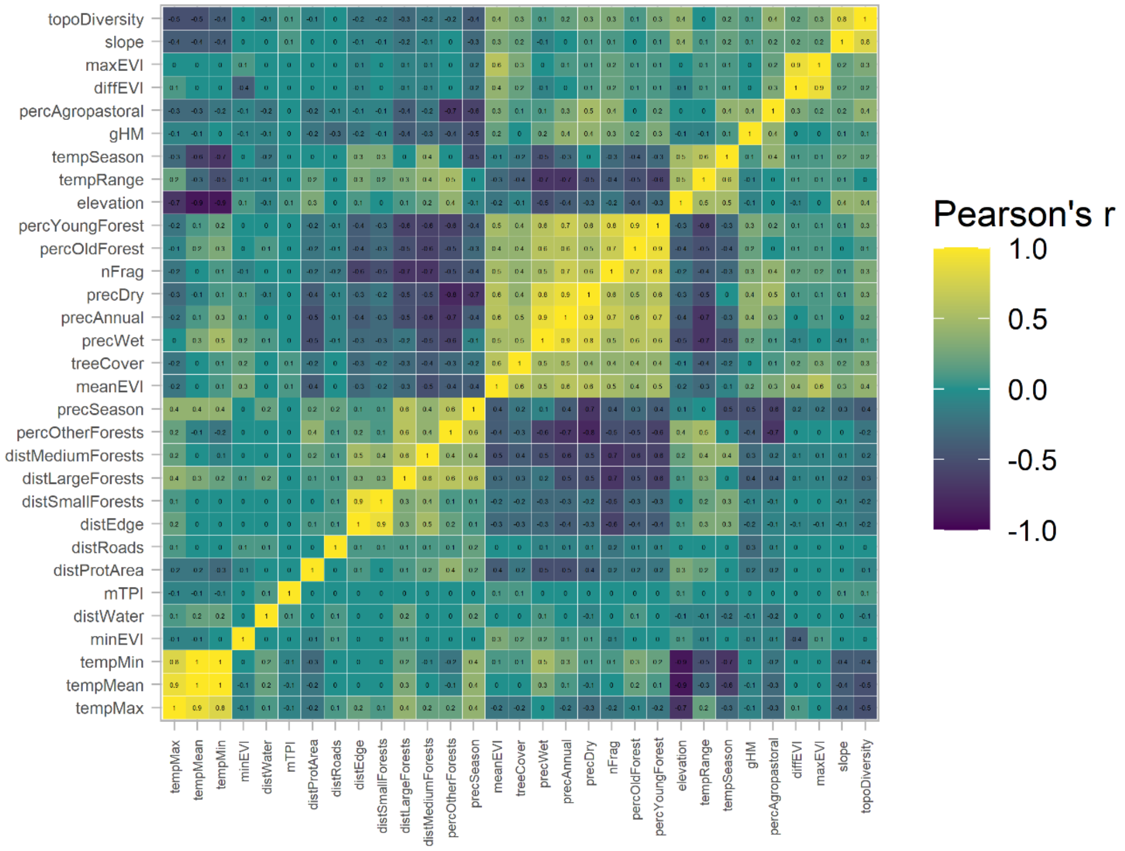


Figure S1. Correlation matrix showing Pearson's r for the 31 pre-defined environmental variables.

Table S3. Variation Inflation Factor (VIF) for the remaining 17 environmental variables with Pearson's < 0.7. For VIF, the remaining variables were continuously removed until none showed VIF > 5.

| Variables | VIF | Variables | VIF |
|---|---|---|---|
| distEdge | 1.395206 | minEVI | 1.171803 |
| distLargeForests | 2.827853 | mTPI | 1.063299 |
| distMediumForests | 2.01613 | percAgropastoral | 1.908332 |
| distProtArea | 1.459872 | percOldForest | 2.447882 |
| distRoads | 1.158977 | precSeason | 3.289526 |
| distWater | 1.161358 | slope | 1.382391 |
| elevation | 1.994298 | tempRange | 1.960882 |
| gHM | 1.773882 | treeCover | 1.824317 |
| maxEVI | 1.365392 | | |

## APPENDIX 3. LIST OF THE PROTECTED AREAS OF THE PERNAMBUCO ENDEMISM CENTER

Table S4. List of protected areas.

| ID | Name | Protection category | Longitude | Latitude | Source |
|---|---|---|---|---|---|
| 1 | Xukuru | Indigenous Area | -36.76935 | -8.32459 | UNEP-WCMC & IUCN (2023) |
| 2 | Kapinawá | Indigenous Area | -37.34713 | -8.61663 | UNEP-WCMC & IUCN (2023) |
| 3 | Fulni-ô | Indigenous Reserve | -37.11164 | -9.11217 | UNEP-WCMC & IUCN (2023) |
| 4 | Xukuru-Kariri | Indigenous Area | -36.62485 | -9.38914 | UNEP-WCMC & IUCN (2023) |
| 5 | Wassu-Cocal | Indigenous Area | -35.71439 | -9.04695 | UNEP-WCMC & IUCN (2023) |
| 6 | Caiçara/Ilha de São Pedro | Indigenous Area | -37.38737 | -9.81895 | UNEP-WCMC & IUCN (2023) |
| 7 | Tingui Botó | Indigenous Reserve | -36.72738 | -9.91975 | UNEP-WCMC & IUCN (2023) |
| 8 | Kariri-Xocó | Indigenous Area | -36.83398 | -10.14106 | UNEP-WCMC & IUCN (2023) |
| 9 | Acaú-Goiana | Extractive Reserve | -34.86146 | -7.55648 | UNEP-WCMC & IUCN (2023) |
| 10 | Barra do Rio Camaratuba | Area of Relevant Ecological Interest | -34.97663 | -6.59774 | UNEP-WCMC & IUCN (2023) |
| 11 | Jenipabu | Environmental Protection Area | -35.21417 | -5.71171 | UNEP-WCMC & IUCN (2023) |
| 12 | Bonfim/Guaraíra | Environmental Protection Area | -35.1735 | -6.08299 | UNEP-WCMC & IUCN (2023) |
| 13 | Ponta do Tubarão | Sustainable Development Reserve | -36.46454 | -5.08996 | UNEP-WCMC & IUCN (2023) |
| 14 | Piquiri-Una | Environmental Protection Area | -35.24754 | -6.37212 | UNEP-WCMC & IUCN (2023) |
| 15 | Catolé e Fernão Velho | Environmental Protection Area | -35.79759 | -9.58277 | UNEP-WCMC & IUCN (2023) |
| 16 | Marituba do Peixe | Environmental Protection Area | -36.40917 | -10.33709 | UNEP-WCMC & IUCN (2023) |
| 17 | Kariri-Xocó | Indigenous Area | -36.83559 | -10.14026 | UNEP-WCMC & IUCN (2023) |
| 18 | Potiguara de Monte-Mor | Indigenous Area | -35.05487 | -6.77675 | UNEP-WCMC & IUCN (2023) |
| 19 | Potiguara | Indigenous Area | -35.14128 | -6.66069 | UNEP-WCMC & IUCN (2023) |
| 20 | Furna Feia | National Park | -37.51106 | -5.05432 | UNEP-WCMC & IUCN (2023) |
| 21 | Mata do Urucu | Wildlife Refuge | -35.25372 | -8.24272 | UNEP-WCMC & IUCN (2023) |
| 22 | Mata da Usina São José | Wildlife Refuge | -35.00174 | -7.8355 | UNEP-WCMC & IUCN (2023) |
| 23 | Mata de Caraúna | Wildlife Refuge | -35.10097 | -8.18649 | UNEP-WCMC & IUCN (2023) |
| 24 | Mata do Contra-Açude | Wildlife Refuge | -35.02191 | -8.23046 | UNEP-WCMC & IUCN (2023) |
| 25 | Serra do Cumaru | Wildlife Refuge | -35.17534 | -8.20428 | UNEP-WCMC & IUCN (2023) |
| 26 | Mata Serra do Cotovelo | Wildlife Refuge | -35.20679 | -8.24632 | UNEP-WCMC & IUCN (2023) |
| 27 | Saltinho | Biological Reserve | -35.18163 | -8.72424 | UNEP-WCMC & IUCN (2023) |
| 28 | Manguezais da Foz do Rio Mamanguape | Area of Relevant Ecological Interest | -34.98884 | -6.79503 | UNEP-WCMC & IUCN (2023) |
| 29 | Piaçabuçu | Environmental Protection Area | -36.3567 | -10.40067 | UNEP-WCMC & IUCN (2023) |

| | | | | | |
|---|---|---|---|---|---|
| 30 | Santa Isabel | Biological Reserve | -36.70948 | -10.6394 | UNEP-WCMC & IUCN (2023) |
| 31 | Pedra Talhada | Biological Reserve | -36.428 | -9.22843 | UNEP-WCMC & IUCN (2023) |
| 32 | Guaribas | Biological Reserve | -35.15736 | -6.71969 | UNEP-WCMC & IUCN (2023) |
| 33 | Potiguara | Indigenous Area | -35.00317 | -6.68897 | UNEP-WCMC & IUCN (2023) |
| 34 | Jacaré de São Domingos | Indigenous Area | -35.08442 | -6.73588 | UNEP-WCMC & IUCN (2023) |
| 35 | Barra do Rio Mamanguape | Environmental Protection Area | -34.96876 | -6.79588 | UNEP-WCMC & IUCN (2023) |
| 36 | Murici | Ecological Station | -35.85004 | -9.2288 | UNEP-WCMC & IUCN (2023) |
| 37 | Catimbau | National Park | -37.34719 | -8.50381 | UNEP-WCMC & IUCN (2023) |
| 38 | Nísia Floresta | National Forest | -35.18228 | -6.08155 | UNEP-WCMC & IUCN (2023) |
| 39 | Açu | National Forest | -36.94732 | -5.57751 | UNEP-WCMC & IUCN (2023) |
| 40 | Restinga de Cabedelo | National Forest | -34.85655 | -7.06405 | UNEP-WCMC & IUCN (2023) |
| 41 | Bicho Homem | Private Reserve of Natural Heritage | -35.72713 | -8.61010 | Carvalho *et al*. (2021) |
| 42 | Jussaral | Private Reserve of Natural Heritage | -35.72713 | -8.61010 | Carvalho *et al*. (2021) |
| 43 | Benedito | Private Reserve of Natural Heritage | -35.58576 | -8.29462 | Carvalho *et al*. (2021) |
| 44 | Engenho Contestado | Private Reserve of Natural Heritage | -35.80112 | -8.84286 | Carvalho *et al*. (2021) |
| 45 | EcoFazenda Morim | Private Reserve of Natural Heritage | -35.20933 | -8.86817 | Carvalho *et al*. (2021) |
| 46 | Fazenda Santa Rita | Private Reserve of Natural Heritage | -35.48577 | -8.69061 | Carvalho *et al*. (2021) |
| 47 | Fazenda Tabatinga | Private Reserve of Natural Heritage | -34.82401 | -7.60474 | Carvalho *et al*. (2021) |
| 48 | Laje Bonita | Private Reserve of Natural Heritage | -36.01678 | -8.80221 | Carvalho *et al*. (2021) |
| 49 | Pedra d'Antas | Private Reserve of Natural Heritage | -35.85588 | -8.69391 | Carvalho *et al*. (2021) |
| 50 | Serro Azul | Private Reserve of Natural Heritage | -35.98936 | -8.13992 | Carvalho *et al*. (2021) |
| 51 | Trapiche | Private Reserve of Natural Heritage | -35.06278 | -8.58014 | Carvalho *et al*. (2021) |
| 52 | Reserva Gulandim | Private Reserve of Natural Heritage | -36.34000 | -9.97700 | Carvalho *et al*. (2021) |
| 53 | Reserva Santa Tereza | Private Reserve of Natural Heritage | -35.97400 | -9.50700 | Carvalho *et al*. (2021) |
| 54 | Fazenda São Pedro | Private Reserve of Natural Heritage | -35.95100 | -9.55500 | Carvalho *et al*. (2021) |
| 55 | Fazenda Rosa do Sol | Private Reserve of Natural Heritage | -35.90500 | -9.83200 | Carvalho *et al*. (2021) |
| 56 | Fazenda Pereira | Private Reserve of Natural Heritage | -36.36130 | -10.25020 | Carvalho *et al*. (2021) |
| 57 | Lula Lobo | Private Reserve of Natural Heritage | -36.35080 | -10.29100 | Carvalho *et al*. (2021) |
| 58 | Vera Cruz | Private Reserve of Natural Heritage | -36.29300 | -9.25600 | Carvalho *et al*. (2021) |
| 59 | Engenho Gargaú | Private Reserve of Natural Heritage | -34.95413 | -7.01558 | Carvalho *et al*. (2021) |
| 60 | Fazenda Pacatuba | Private Reserve of Natural Heritage | -35.15641 | -7.04635 | Carvalho *et al*. (2021) |
| 61 | Reserva Calaça | Private Reserve of Natural Heritage | -36.22294 | -8.71872 | Carvalho *et al*. (2021) |
| 62 | Serra do Contente | Private Reserve of Natural Heritage | -35.55289 | -8.26137 | Carvalho *et al*. (2021) |

| | | | | | |
|---|---|---|---|---|---|
| 63 | Nossa Senhora do Oiteiro de Maracaípe | Private Reserve of Natural Heritage | -35.01678 | -8.52391 | Carvalho *et al*. (2021) |
| 64 | Frei Caneca | Private Reserve of Natural Heritage | -35.84439 | -8.71929 | Carvalho *et al*. (2021) |
| 65 | Reserva Cabanos | Private Reserve of Natural Heritage | -36.01042 | -8.49913 | Carvalho *et al*. (2021) |
| 66 | Fazenda Santa Beatriz do Carnijó | Private Reserve of Natural Heritage | -35.07890 | -8.14150 | Carvalho *et al*. (2021) |
| 67 | Mata Estrela | Private Reserve of Natural Heritage | -35.00043 | -6.40460 | Carvalho *et al*. (2021) |
| 68 | Dunas Douradas | Private Reserve of Natural Heritage | -35.23917 | -5.62564 | Carvalho *et al*. (2021) |
| 69 | Mata da Bela | Private Reserve of Natural Heritage | -35.11431 | -6.42253 | Carvalho *et al*. (2021) |
| 70 | Triunfo | Private Reserve of Natural Heritage | -35.29175 | -9.05136 | Carvalho *et al*. (2021) |
| 71 | Tobogã | Private Reserve of Natural Heritage | -35.77266 | -9.60078 | Carvalho *et al*. (2021) |
| 72 | Santa Fé | Private Reserve of Natural Heritage | -36.44521 | -9.52089 | Carvalho *et al*. (2021) |
| 73 | Placas | Private Reserve of Natural Heritage | -35.60659 | -9.43190 | Carvalho *et al*. (2021) |
| 74 | Cachoeira | Private Reserve of Natural Heritage | -36.44492 | -9.53388 | Carvalho *et al*. (2021) |
| 75 | Cachoeira | Private Reserve of Natural Heritage | -35.25073 | -8.97370 | Carvalho *et al*. (2021) |
| 76 | Bosque | Private Reserve of Natural Heritage | -35.23164 | -8.94939 | Carvalho *et al*. (2021) |
| 77 | Aldeia Verde | Private Reserve of Natural Heritage | -35.69592 | -9.57279 | Carvalho *et al*. (2021) |
| 78 | Planalto | Private Reserve of Natural Heritage | -36.35151 | -10.16927 | Carvalho *et al*. (2021) |
| 79 | Madeiras | Private Reserve of Natural Heritage | -36.33726 | -9.87378 | Carvalho *et al*. (2021) |
| 80 | Estrela do Sul | Private Reserve of Natural Heritage | -35.70765 | -8.93338 | Carvalho *et al*. (2021) |
| 81 | Porto Alegre | Private Reserve of Natural Heritage | -35.68117 | -8.93055 | Carvalho *et al*. (2021) |
| 82 | Papa Mel | Private Reserve of Natural Heritage | -35.67948 | -8.93047 | Carvalho *et al*. (2021) |
| 83 | Porto Seguro | Private Reserve of Natural Heritage | -35.51017 | -9.09886 | Carvalho *et al*. (2021) |
| 84 | Canadá | Private Reserve of Natural Heritage | -36.37585 | -9.46256 | Carvalho *et al*. (2021) |
| 85 | Vila d'Água | Private Reserve of Natural Heritage | -35.94150 | -9.29038 | Carvalho *et al*. (2021) |
| 86 | Boa Sorte | Private Reserve of Natural Heritage | -35.92943 | -9.18714 | Carvalho *et al*. (2021) |
| 87 | Osvaldo Timóteo | Private Reserve of Natural Heritage | -36.03271 | -9.02953 | Carvalho *et al*. (2021) |
| 88 | Santa Maria | Private Reserve of Natural Heritage | -35.85299 | -9.35532 | Carvalho *et al*. (2021) |
| 89 | Mata do Cedro | Private Reserve of Natural Heritage | -35.90690 | -9.52350 | Carvalho *et al*. (2021) |
| 90 | Serra d'Água | Private Reserve of Natural Heritage | -35.57598 | -9.11430 | Carvalho *et al*. (2021) |
| 91 | Garabu | Private Reserve of Natural Heritage | -35.58037 | -9.28120 | Carvalho *et al*. (2021) |
| 92 | Saint Michel 1 | Private Reserve of Natural Heritage | -35.87679 | -9.80366 | Carvalho *et al*. (2021) |
| 93 | Santa Cristina | Private Reserve of Natural Heritage | -35.95204 | -9.79145 | Carvalho *et al*. (2021) |
| 94 | Sereno | Private Reserve of Natural Heritage | -35.38060 | -9.09754 | Carvalho *et al*. (2021) |
| 95 | Quebra Carro | Private Reserve of Natural Heritage | -36.10873 | -9.63123 | Carvalho *et al*. (2021) |

| | | | | | |
|---|---|---|---|---|---|
| 96 | Saint Michel 2 | Private Reserve of Natural Heritage | -35.86372 | -9.79874 | Carvalho *et al*. (2021) |
| 97 | Baixa Grande | Private Reserve of Natural Heritage | -36.18787 | -9.70980 | Carvalho *et al*. (2021) |
| 98 | Conceição Lyra I | Private Reserve of Natural Heritage | -36.45797 | -10.20955 | Carvalho *et al*. (2021) |
| 99 | Saint Michel 3 | Private Reserve of Natural Heritage | -35.86501 | -9.79936 | Carvalho *et al*. (2021) |
| 100 | Conceição Lyra IV | Private Reserve of Natural Heritage | -36.45947 | -10.19935 | Carvalho *et al*. (2021) |
| 101 | Salvador Lyra | Private Reserve of Natural Heritage | -36.02396 | -9.75347 | Carvalho *et al*. (2021) |
| 102 | Boca do Rio | Private Reserve of Natural Heritage | -35.98500 | -9.78628 | Carvalho *et al*. (2021) |
| 103 | Riacho Seco | Private Reserve of Natural Heritage | -36.22481 | -10.09272 | Carvalho *et al*. (2021) |
| 104 | Conceição Lyra II | Private Reserve of Natural Heritage | -36.45263 | -10.19971 | Carvalho *et al*. (2021) |
| 105 | Oriente | Private Reserve of Natural Heritage | -35.45344 | -9.01184 | Carvalho *et al*. (2021) |
| 106 | Conceição Lyra III | Private Reserve of Natural Heritage | -36.44832 | -10.16385 | Carvalho *et al*. (2021) |
| 107 | Pindoba | Private Reserve of Natural Heritage | -35.99463 | -9.78298 | Carvalho *et al*. (2021) |
| 108 | Apolinário | Private Reserve of Natural Heritage | -35.63923 | -9.39100 | Carvalho *et al*. (2021) |
| 109 | Olho d'Água | Private Reserve of Natural Heritage | -35.97399 | -9.78930 | Carvalho *et al*. (2021) |

## References


CARVALHO, C. S. et al. Environmental heterogeneity and sampling relevance areas in an Atlantic Forest endemism region. Perspectives in Ecology and Conservation, v. 19, n. 3, p. 311-318, 2021.

UNEP-WCMC; IUCN. Protected Planet: The World Database on Protected Areas (WDPA) and World Database on Other Effective Area-based Conservation Measures (WD-OECM). Cambridge: UNEP-WCMC, 2023. Disponível em: < www.protectedplanet.net>

## APPENDIX 4. CROSS-TAXA EVALUATION OF THE ENSEMBLE SPECIES DISTRIBUTION MODELS

Table S5. Performance evaluation for GLM, GBM, CTA, ANN, MAXENT, and Ensemble (EM) based on True Skill Statistic (TSS) for 30 bird taxa of the Pernambuco Endemism Center (PEC), in northeastern Brazilian Atlantic Forest.

| Taxa | GLM Mean TSS | GBM Mean TSS | CTA Mean TSS | ANN Mean TSS | MAXENT Mean TSS | EM Weighted Mean TSS |
|---|---|---|---|---|---|---|
| *Automolus lammi* | 0.79 | 0.84 | 0.67 | 0.68 | 0.60 | 0.97 |
| *Caryothraustes brasiliensis* (NE pop.) | 0.65 | 0.66 | 0.66 | 0.56 | 0.83 | 0.98 |
| *Cercomacroides laeta sabinoi* | 0.74 | 0.85 | 0.71 | 0.62 | 0.82 | 0.97 |
| *Conopophaga cearae* | 0.66 | 0.70 | 0.49 | 0.55 | 0.56 | 0.96 |
| *Conopophaga melanops nigrifrons* | 0.69 | 0.72 | 0.53 | 0.59 | 0.74 | 0.91 |
| *Dendrocincla taunayi* | 0.38 | 0.84 | 0.74 | 0.45 | 0.82 | 1.00 |
| *Hemithraupis flavicollis melanoxantha* | 0.59 | 0.85 | 0.72 | 0.67 | 0.93 | 0.99 |
| *Hemitriccus griseipectus naumburgae* | 0.80 | 0.84 | 0.66 | 0.72 | 0.84 | 0.92 |
| *Hemitriccus mirandae* | 0.40 | 0.73 | 0.63 | 0.34 | 0.85 | 0.98 |
| *Iodopleura pipra leucopygia* | 0.29 | 0.71 | 0.27 | 0.28 | 0.81 | 1.00 |
| *Leptodon forbesi* | 0.78 | 0.82 | 0.68 | 0.57 | 0.75 | 0.92 |
| *Megascops alagoensis* | 0.70 | 0.78 | 0.61 | 0.59 | 0.75 | 1.00 |
| *Momotus momota marcgravianus* | 0.52 | 0.67 | 0.68 | 0.51 | 0.89 | 0.98 |
| *Myrmoderus ruficauda soror* | 0.79 | 0.83 | 0.69 | 0.62 | 0.75 | 0.98 |
| *Myrmotherula snowi* | 0.70 | 0.89 | 0.84 | 0.66 | 0.94 | 0.99 |
| *Penelope superciliaris alagoensis* | 0.63 | 0.62 | 0.36 | 0.37 | 0.63 | 0.92 |
| *Phylloscartes ceciliae* | 0.84 | 0.88 | 0.79 | 0.66 | 0.85 | 0.98 |
| *Picumnus pernambucensis* | 0.77 | 0.83 | 0.72 | 0.58 | 0.74 | 0.97 |
| *Platyrinchus mystaceus niveigularis* | 0.77 | 0.79 | 0.48 | 0.48 | 0.79 | 0.92 |
| *Pyriglena pernambucensis* | 0.70 | 0.83 | 0.77 | 0.64 | 0.85 | 0.91 |
| *Schiffornis turdina intermedia* | 0.76 | 0.78 | 0.75 | 0.63 | 0.87 | 0.91 |
| *Synallaxis infuscata* | 0.75 | 0.76 | 0.63 | 0.56 | 0.48 | 0.86 |

| | | | | | | |
|---|---|---|---|---|---|---|
| *Tangara cyanocephala cearensis* | 0.43 | 0.83 | 0.54 | 0.44 | 0.87 | 0.90 |
| *Tangara fastuosa* | 0.79 | 0.81 | 0.67 | 0.52 | 0.79 | 0.87 |
| *Terenura sicki* | 0.84 | 0.88 | 0.84 | 0.68 | 0.73 | 0.94 |
| *Thalurania watertonii* | 0.79 | 0.80 | 0.67 | 0.58 | 0.81 | 0.84 |
| *Thamnophilus aethiops distans* | 0.79 | 0.81 | 0.60 | 0.47 | 0.79 | 0.98 |
| *Thamnophilus caerulescens pernambucensis* | 0.70 | 0.72 | 0.51 | 0.49 | 0.77 | 0.97 |
| *Xenops minutus alagoanus* | 0.73 | 0.80 | 0.59 | 0.64 | 0.77 | 0.80 |
| *Xiphorhynchus atlanticus* | 0.81 | 0.85 | 0.69 | 0.66 | 0.70 | 0.91 |

Table S6. Ensemble models evaluation based on Receiver Operating-Characteristic (ROC) and True Skill Statistic (TSS) for 30 bird taxa of the Pernambuco Endemism Center (PEC), in northeastern Brazilian Atlantic Forest.

| Taxa | ROC Test. data | ROC Cut. | ROC Sens. | ROC Spec. | TSS Test. data | TSS Cut. | TSS Sens. | TSS Spec. |
|---|---|---|---|---|---|---|---|---|
| *Automolus lammi* | 1 | 439.5 | 100 | 96.97 | 0.97 | 439 | 100 | 96.96 |
| *Caryothraustes brasiliensis* (NE pop.) | 1 | 538.5 | 100 | 98.03 | 0.98 | 531 | 100 | 97.91 |
| *Cercomacroides laeta sabinoi* | 1 | 336.5 | 100 | 97.13 | 0.97 | 330 | 100 | 97 |
| *Conopophaga cearae* | 0.99 | 493.5 | 100 | 96.09 | 0.96 | 490 | 100 | 95.97 |
| *Conopophaga melanops nigrifrons* | 0.99 | 382.5 | 100 | 91.68 | 0.91 | 376 | 100 | 91.41 |
| *Dendrocincla taunayi* | 1 | 471.5 | 100 | 99.66 | 1 | 470 | 100 | 99.63 |
| *Hemithraupis flavicollis melanoxantha* | 1 | 453.5 | 100 | 98.84 | 0.99 | 449 | 100 | 98.79 |
| *Hemitriccus griseipectus naumburgae* | 0.99 | 327.5 | 98.3 | 93.38 | 0.92 | 326 | 98.28 | 93.33 |
| *Hemitriccus mirandae* | 1 | 494.5 | 100 | 98.51 | 0.98 | 487 | 100 | 98.44 |
| *Iodopleura pipra leucopygia* | 1 | 690.5 | 100 | 99.97 | 1 | 690 | 100 | 99.97 |
| *Leptodon forbesi* | 0.99 | 323.5 | 100 | 91.6 | 0.92 | 319 | 100 | 91.45 |
| *Megascops alagoensis* | 1 | 682.5 | 100 | 99.97 | 1 | 683 | 100 | 99.97 |
| *Momotus momota marcgravianus* | 1 | 351.5 | 100 | 98.12 | 0.98 | 345 | 100 | 98.02 |
| *Myrmoderus ruficauda soror* | 1 | 520.5 | 100 | 97.7 | 0.98 | 513 | 100 | 97.57 |

| | | | | | | | | |
|---|---|---|---|---|---|---|---|---|
| *Myrmotherula snowi* | 1 | 581 | 100 | 99.4 | 0.99 | 574 | 100 | 99.37 |
| *Penelope superciliaris alagoensis* | 0.99 | 469.5 | 100 | 91.73 | 0.92 | 467 | 100 | 91.49 |
| *Phylloscartes ceciliae* | 1 | 399.5 | 100 | 97.87 | 0.98 | 393 | 100 | 97.83 |
| *Picumnus pernambucensis* | 0.99 | 287.5 | 100 | 96.46 | 0.96 | 282 | 100 | 96.22 |
| *Platyrinchus mystaceus niveigularis* | 0.98 | 212.5 | 100 | 92.98 | 0.93 | 209 | 100 | 92.49 |
| *Pyriglena pernambucensis* | 0.98 | 184.5 | 95.5 | 95.98 | 0.91 | 184 | 95.46 | 95.88 |
| *Schiffornis turdina intermedia* | 0.95 | 211.5 | 100 | 91.41 | 0.91 | 209 | 100 | 91.04 |
| *Synallaxis infuscata* | 0.96 | 288.5 | 94.4 | 90.52 | 0.85 | 285 | 94.38 | 90.12 |
| *Tangara cyanocephala cearensis* | 0.98 | 167.5 | 100 | 95.63 | 0.96 | 166 | 100 | 95.54 |
| *Tangara fastuosa* | 0.97 | 198.5 | 98.4 | 87.67 | 0.86 | 194 | 98.41 | 87.09 |
| *Terenura sicki* | 0.98 | 238.5 | 97.6 | 96.47 | 0.94 | 233 | 97.56 | 96.25 |
| *Thalurania watertonii* | 0.95 | 182.5 | 100 | 87.84 | 0.88 | 182 | 100 | 87.78 |
| *Thamnophilus aethiops distans* | 0.99 | 303.5 | 100 | 98.16 | 0.98 | 302 | 100 | 98.07 |
| *Thamnophilus caerulescens pernambucensis* | 0.98 | 263.5 | 100 | 95.02 | 0.95 | 263 | 100 | 94.93 |
| *Xenops minutus alagoanus* | 0.94 | 185.5 | 100 | 83.19 | 0.83 | 183 | 100 | 82.84 |
| *Xiphorhynchus atlanticus* | 0.98 | 167.5 | 100 | 92.4 | 0.92 | 166 | 100 | 92.21 |

**APPENDIX 4. ENDEMIC AND THREATENED FOREST-DWELLING BIRDS DISTRIBUTION ACROSS THE PERNAMBUCO ENDEMISM CENTER**

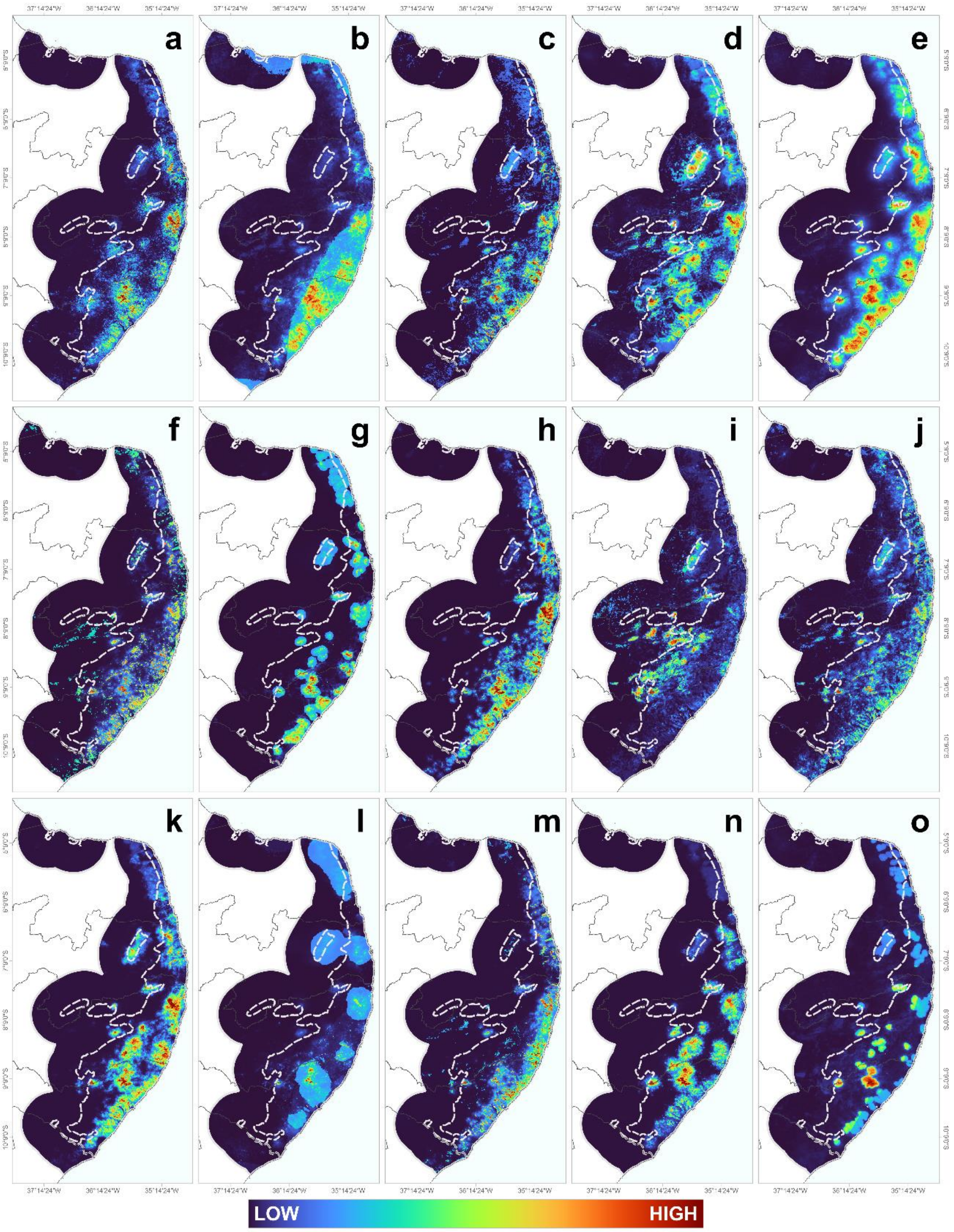


Figure S2. Prediction of distribution for 30 endemic and threatened forest-dwelling birds across the Pernambuco Endemism Center, in northeastern Brazil. a = *Automolus lammi*; b = *Caryothraustes brasiliensis* (NE pop.); c = *Cercomacroides laeta sabinoi*; d = *Conopophaga cearae*; e = *Conopophaga melanops nigrifrons*; f = *Dendrocincla taunayi*; g = *Hemithraupis flavicollis melanoxantha*; h = *Hemitriccus griseipectus naumburgae*; i = *Hemitriccus mirandae*; j = *Iodopleura pipra leucopygia*; k = *Leptodon forbesi*; l =

*Megascops alagoensis*; m = *Momotus momota marcgravianus*; n = *Myrmoderus ruficauda soror*; o = *Myrmotherula snowi*; p = *Penelope superciliaris alagoensis*; q = *Phylloscartes ceciliae*; r = *Picumnus pernambucensis*; s = *Platyrinchus mystaceus niveigularis*; t = *Pyriglena pernambucensis*; u = *Schiffornis turdina intermedia*; v = *Synallaxis infuscate*; w = *Tangara cyanocephala cearensis*; x = *Tangara fastuosa*; y = *Terenura sicki*; z = *Thalurania watertonii*; aa = *Thamnophilus aethiops distans*; ab = *Thamnophilus caerulescens pernambucensis*; ac = *Xenops minutus alagoanus*; ad = *Xiphorhynchus atlanticus*.

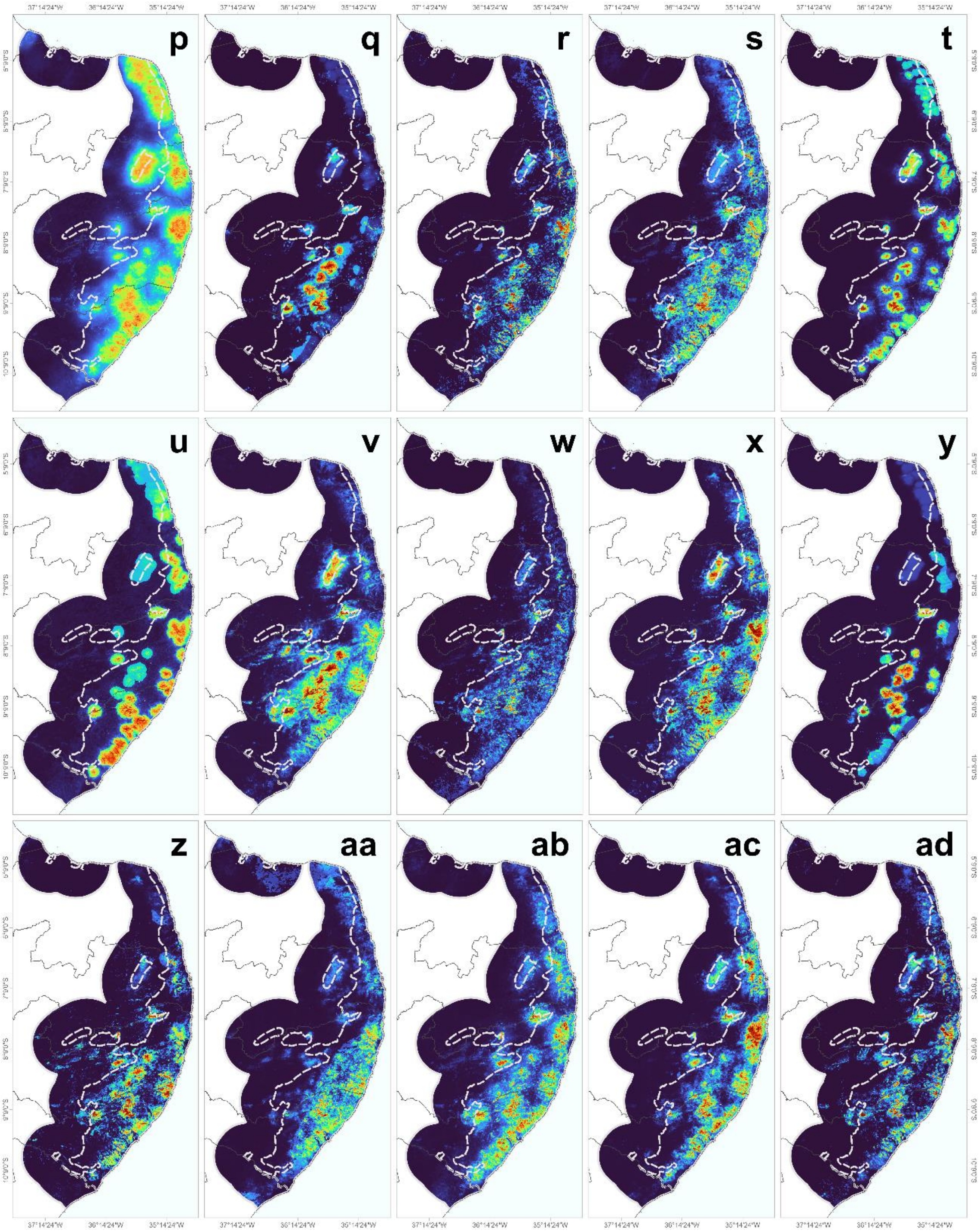


Figure S2. (continued)
